# Unraveling blunt-end RNA binding and ATPase-driven translocation activities of the RIG-I family helicase LGP2

**DOI:** 10.1093/nar/gkad1106

**Published:** 2023-11-28

**Authors:** Kuan-Ying Lee, Candice Craig, Smita S Patel

**Affiliations:** Department of Biochemistry and Molecular Biology, Rutgers University, Robert Wood Johnson Medical School, Piscataway, NJ 08854, USA; Graduate School of Biomedical Sciences at the Robert Wood Johnson Medical School of Rutgers University, 08854, USA; Department of Biochemistry and Molecular Biology, Rutgers University, Robert Wood Johnson Medical School, Piscataway, NJ 08854, USA; Graduate School of Biomedical Sciences at the Robert Wood Johnson Medical School of Rutgers University, 08854, USA; Department of Biochemistry and Molecular Biology, Rutgers University, Robert Wood Johnson Medical School, Piscataway, NJ 08854, USA

## Abstract

The RIG-I family helicases, comprising RIG-I, MDA5 and LGP2, are cytoplasmic RNA sensors that trigger an antiviral immune response by specifically recognizing foreign RNAs. While LGP2 lacks the signaling domain necessary for immune activation, it plays a vital role in regulating the RIG-I/MDA5 signaling pathway. In this study, we investigate the mechanisms underlying this regulation by examining the oligomeric state, RNA binding specificity, and translocation activity of human LGP2 and the impact of ATPase activity. We show that LGP2, like RIG-I, prefers binding blunt-ended double-stranded (ds) RNAs over internal dsRNA regions or RNA overhangs and associates with blunt-ends faster than with overhangs. Unlike RIG-I, a 5′-triphosphate (5′ppp), Cap0, or Cap1 RNA-end does not influence LGP2’s RNA binding affinity. LGP2 hydrolyzes ATP in the presence of RNA but at a 5–10 fold slower rate than RIG-I. Nevertheless, LGP2 uses its ATPase activity to translocate and displace biotin-streptavidin interactions. This activity is significantly hindered by a methylated RNA patch, particularly on the 3′-strand, suggesting a 3′-strand tracking mechanism like RIG-I. The preference of LGP2 for blunt-end RNA binding, its insensitivity to Cap0/Cap1 modification, and its translocation/protein displacement ability have substantial implications for how LGP2 regulates the RNA sensing process by MDA5/RIG-I.

## Graphical Abstract

**Graphical Abstract:**
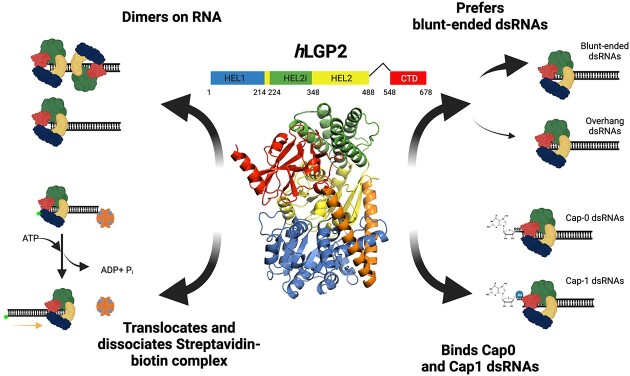

## Introduction

The innate immune system serves as the first line of defense against pathogen infections, and the RIG-I-like receptors (RLRs) play a crucial role in this process (1–3). RLRs belong to the DExD/H family of ATPase/helicase and consist of three members: retinoic acid-inducible gene I (RIG-I or DDX58), melanoma differentiation-associated gene 5 (MDA5 or IFIH1), and laboratory of genetics and physiology 2 (LGP2 or DHX58). These receptors possess distinct RNA binding properties, enabling them to recognize a wide range of viruses. RIG-I recognizes blunt-ended double-stranded (ds) RNAs with a 5′-triphosphate (5′ppp) and 5′PP (diphosphate), while MDA5 recognizes long dsRNA regions (4–8). These RNA features are commonly found in viral RNA genomes and replication intermediates generated during viral infections, and detecting these foreign RNAs is critical for activating the RIG-I/MDA5 signaling pathway for interferon-mediated antiviral response. LGP2, the smallest member of the RLR family, is pivotal in regulating the signaling pathway through positive and negative regulation of MDA5 and RIG-I, respectively (9–12).

Studies show that LGP2 inhibits RIG-I, and at low concentrations, LGP2 activates MDA5 while inhibiting at higher concentrations (1,3,13–16). While the exact mechanism of RIG-I inhibition and MDA5 activation is not fully established, the current model suggests that LGP2 activates MDA5 by nucleating its multimerization on long dsRNA (9,17). However, overexpression of LGP2 leads to inhibition through feedback regulation involving interference with the ubiquitination steps in the RIG-I/MDA5-activated signaling pathway downstream of RNA binding (18,19). This feedback inhibition mechanism is essential for preventing excessive immune responses by the RLRs. In addition to regulating the viral RNA response activities of RIG-I and MDA5, LGP2 expression affects many other cellular processes, including CD8^+^ T cell survival, conferring cancer cells radioresistant or sensitive to ionizing radiation therapy depending on where LGP2 is expressed, and inhibiting the Dicer-mediated interference (RNAi) pathway (20,21).

**Figure 1.**
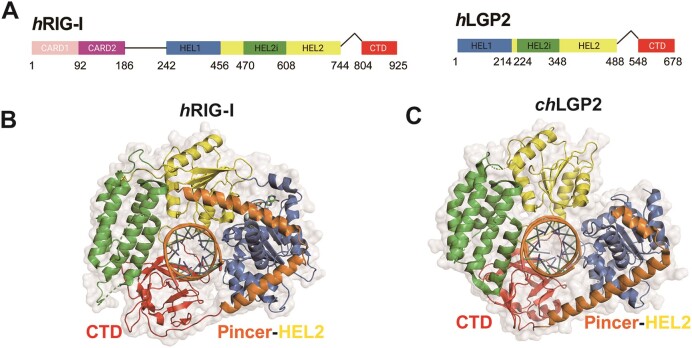
Structures of LGP2 and RIG-I. (**A**) Domain structure of human RIG-I (hRIG-I) and LGP2 (hLGP2). Domain colors are blue (HEL1), green (HEL2i), yellow (HEL2) and red (CTD). (B, C) Head-end cartoon view of hRIG-I-dsRNA-ADP:BeF_3_ complex (PDB: 5E3H) (**B**) compared with chicken LGP2 (chLGP2)-dsRNA-ADP:AlF_4_ complex (PDB: 5JBJ) (**C**).

The RLR family members possess a conserved superfamily 2 helicase structure comprising a core helicase domain with HEL1 and HEL2 subdomains interrupted by an insertion domain, HEL2i (Figure 1A) (6,22,23). The ATPase active site is located at the interface of HEL1 and HEL2, responding to RNA binding by promoting ATP hydrolysis. RIG-I and MDA5 also contain tandem N-terminal caspase activation and recruitment domains (CARDs) connected to HEL1 via an acidic intrinsically disordered linker that autoinhibits RIG-I CARDs and prevents the helicase domain from indiscriminately binding RNAs (24). The conformational changes caused by viral RNA binding expose the CARDs, enabling their interactions with the downstream adapter proteins, such as ubiquitin chains and MAVS (25). LGP2 lacks these signaling CARDs and the associated intrinsically disordered linker. Consequently, LGP2 can bind to dsRNA but cannot activate the RIG-I/MDA5 signaling pathway.

All three RLRs have a common feature—a C-terminal domain (CTD) that plays a crucial role in recognizing specific RNA features. For example, in the case of RIG-I and LGP2, their respective CTDs bind to the dsRNA end, while MDA5’s CTD binds to the RNA backbone (26–28). RIG-I’s CTD recognizes and binds to the 5′ppp and blunt-end regions of RNA and can tolerate bulky adducts such as the m7G cap (Cap 0) (29) and metabolite caps at the 5′ppp end (30). However, 2′-*O*-methylation on the ribose of the first base pair from the 5′-end (Cap 1) hinders RIG-I binding and immune activation (29,31). It is unknown whether LGP2 shares similar RNA preferences as RIG-I or possesses distinct RNA recognition characteristics. In RIG-I, the CTD is linked to the helical pincer through a flexible linker (Figure 1B), allowing the CTD to survey the RNAs and find the 5′ppp blunt-end (32). Once bound to the 5′ppp blunt-end, the CTD in RIG-I assists in loading the RNA into the helicase domain, resulting in additional conformational changes involving full engagement with the adjoining ∼10 bp of RNA stem. The CTD-pincer linkage in LGP2 is more constrained than RIG-I (33), as two extra helical turns replace a portion of the flexible linker (Figure 1C). It remains unknown how this structural difference affects LGP2’s RNA surveillance and binding function and broadly how the RNA binding and ATPase activities of LGP2 regulate its multifaceted function.

To gain further insights into the specificity of RNA binding and the impact of ATPase activity, this study focuses on the quantitative characterization of recombinant human LGP2 protein purified from bacterial and insect cell expression systems. We use biochemical and biophysical approaches to determine the RNA binding specificity of LGP2 and show its preference for blunt-ended RNAs over other RNA ends, such as 5′- or 3′-overhangs or internal regions in dsRNA. In addition, we show that the Cap0/Cap1 modifications do not affect the LGP2’s RNA binding affinity. Our studies also show that LGP2 binds to dsRNA as either a monomer or a dimer but does not form higher-order oligomers like RIG-I and MDA5. Like RIG-I, dsRNA binding stimulates the ATPase activity of LGP2 and drives the translocation of LGP2 on dsRNA. The findings regarding the RNA binding specificity, dsRNA translocation, and ATPase activities of LGP2 have implications for regulating the RNA sensing mechanisms of RIG-I and MDA5.

## Materials and methods

### Nucleic acid substrates

The HP and ds27 RNA strands were chemically synthesized and HPLC purified by TriLink BioTechnologies or Horizon Discovery. As described previously (30), the 5′ppp containing strand of the ds39 RNA was made by *in vitro* transcription. Mass spectrometry and HPLC analysis were performed to assess the purity of the synthetic RNAs. The lyophilized RNA samples were reconstituted in 20 mM potassium phosphate buffer (pH 7.0). The RNA concentration was determined by measuring the absorbance at 260 nm using the NanoDrop spectrophotometer and calculating the concentration based on the extinction coefficients. Duplex RNA was prepared by combining complementary single-stranded RNAs in a 1:1.1 ratio (fluorescent: nonfluorescent ratio, where applicable). The mixture was heated to 95°C for 3–5 min and slowly cooled to 4°C. The sequences of all RNAs used in this study are provided in Supplemental Table S5.

### LGP2 baculovirus generation

Full-length human LGP2 (1–678) with a TEV cleavable 6xHis-tag was cloned into the baculovirus genome using the shuttle vector pBacPak9 (Takara). Co-transfection of pBacPak9 containing full-length human LGP2 and linear pBacPak6 (Bsu36 I digest, Takara), which contains the necessary genome for baculovirus construction, was performed in adhering Sf9 insect cells using Bacfectin (Takara). The supernatant from the adhering Sf9 insect cells was harvested 5 days after transfection to obtain the P_0_ virus. About 1 ml of the P_0_ virus was added to 250 ml of cells at 1 × 10^6^ cells/ml for a 5-day amplification to obtain the P_1_ virus. The P_1_ virus was stored at 4°C for viral titer determination (Expression systems, titer service).

For protein expression, 400 ml of Sf-900 II SFM media in a 1-liter flask were seeded at a density of 3 × 10^5^ cells/ml and grown for 2–3 days at 26–28°C to reach a density of about 1 × 10^6^ cells/ml. Subsequently, the E64 protease inhibitor was added to the cells at a concentration of 1 mg/l (Sigma) along with the P_1_ virus at an MOI of 1. The cells were harvested after 72 h.

### Protein purification

#### LGP2 (Insect cell expressed)

The Sf9 cell pellet (10 g) was resuspended in lysis buffer (50 mM Na_3_PO_4_ (pH 8), 300 mM NaCl, 5 mM MgCl_2_, 0.5% (v/v) NP-40, 0.1% (v/v) Triton X-100, 10% (v/v) glycerol, EDTA-free protease inhibitor tablet (Roche), and 2 mM DTT). The cells were lysed by gently stirring the suspension at 4°C for 2 h. After centrifugation at 31000 g for 30 minutes, the supernatant was subjected to nickel (Ni^2+^) affinity chromatography using a HisTrap HP column (Cytiva). The eluted protein was then treated with TEV protease to remove the His-tag and further purified using a heparin column (HiTrap heparin HP affinity column). Finally, the protein was purified to homogeneity by passing it through a Superdex 200 gel filtration column (Cytiva) using gel filtration buffer (50 mM HEPES, 150 mM NaCl, 0.5 mM TCEP and 5% (v/v) glycerol). The purified protein was snap-frozen in liquid nitrogen and stored at –80°C.

#### LGP2 (*E. coli* expressed)

Full-length human LGP2 protein with a 6xHistidine SUMO tag was expressed in *E. coli* strain Rosetta (DE3) (Novagen). Cell growth and lysis were performed following the published RIG-I purification protocol (24). Cells were lysed as described above for the insect cell expressed LGP2. The soluble fraction of LGP2 was purified from the cell lysate using a HisTrap HP column (Cytiva), Heparin Sepharose column, and Ulp1 protease digestion to cleave the 6xHis-SUMO tag, followed by a second heparin column. A small protein sample was run through a Superose 6 gel filtration column to assess its oligomeric state (Cytiva). The purified protein was snap-frozen in liquid nitrogen and stored at -80°C.Cytiva). The purified protein was snap-frozen in liquid nitrogen and stored at –80°C.

### Electrophoretic mobility shift assays (EMSA)

#### Biotinylated 5′ppp or 5′ Ovg ds39 RNAs EMSA

EMSA assays were conducted by incubating RIG-I or LGP2 with fluorophore-tagged dsRNA at 4°C in Buffer A (comprising 50 mM MOPS, pH 7.4, 0.05 mM BSA, 5 mM DTT, 5 mM MgCl_2_, 0.01% Tween20) for 60 min. The EMSA reactions utilized a range of RIG-I or LGP2 concentrations (as indicated in the legend) at 1.5 to 12-fold molar excess relative to the concentration of fluorophore-tagged biotinylated dsRNA (25 nM). The reactions were supplemented with 2 mM ATP, as specified in the figure legends. Subsequently, loading buffer (comprising 1.5% Ficoll 400 in Tris-borate buffer, pH 8.0) was added to the samples, which were then loaded onto a 4–20% gradient native polyacrylamide gel in TBE buffer (Invitrogen). The gels were scanned at 532 nm using a Typhoon FLA 9500 laser-based scanner (GE) and quantified using ImageQuant software.

#### HP RNAs EMSA

The EMSA reactions were carried out as above utilizing equal or double molar concentrations (20, 40 nM) of RIG-I or LGP2 and the fluorophore-tagged dsRNA (20 nM) and Buffer A supplemented with 100 mM NaCl. Subsequently, the reactions were loaded onto a 20% native polyacrylamide gel in TBE buffer (Invitrogen).

#### Monovalent streptavidin-biotin conjugated 5′ppp, Cap0, and Cap1 ds27 RNAs EMSA

The RNAs (20 nM) were treated with a 1.5-fold molar excess of monovalent streptavidin (provided by Dr. Mark Howarth, University of Oxford) at 4°C in Buffer A for 30 min. The EMSA reactions used a 3-fold molar excess of RIG-I or LGP2 (60 nM) incubated with RNAs at 4°C in Buffer A for 60 min before loading onto a 4–20% gradient native polyacrylamide gel in TBE buffer (Invitrogen).

#### Monovalent streptavidin-desthiobiotin conjugated 5′ppp ds39 EMSA

A 3-fold molar excess of monovalent streptavidin was added to the fluorophore-tagged desthiobiotinylated ds39 RNA (25 nM) at 4°C in Buffer A for 30 min. The EMSA reactions used a range of RIG-I or LGP2 concentrations (as indicated in the legend) from 0.75- to 12-fold molar excess relative to RNA. The samples were loaded onto a 4–20% gradient native polyacrylamide gel in TBE buffer (Invitrogen).

### RNA *K*_D_ measurements using fluorescence polarization titration

The serially diluted LGP2 protein solution was mixed with fluorescein-labeled RNA (Horizon, HPLC purified) at a concentration of 10 nM in buffer A. Fluorescence intensities were measured on the TECAN Spark plate reader with an excitation wavelength of 485 nm (20 nm bandwidth) and an emission wavelength of 535 nm (20 nm bandwidth). The polarization (P) was calculated from the parallel and perpendicular polarized light emission intensities (I) using the equation: [$P = \frac{{( {I\| - I \bot } )}}{{( {I\| + I \bot } )}}$. The millipolarization (mP) of free RNA was subtracted from the protein-bound values and plotted against protein concentration. The binding titration curves were fitted to the equation [$mP = \frac{{{B_{max}} \cdot x}}{{{K_D} + x}}$] using GraphPad Prism 9.0. Here, *B*_max_ is the mP amplitude of the protein-bound RNA complex. *x* is the protein concentration, and *K*_D_ is the equilibrium dissociation constant.

### Kinetics of RNA binding: *k*_on_ and *k*_off_ rate measurements

The RNA off-rates (*k*_off_) were measured at 25°C using a stopped-flow instrument (Auto-SF 120, Kintek Corp, Austin, TX). A mixture of fluorophore-labeled dsRNA, LGP2 and 100 mM NaCl in Buffer A (pre-incubated at 25°C for 10 minutes) was mixed with a 10-fold excess of an RNA trap in 100 mM NaCl in Buffer A (unlabeled 5′-ppp ds28 palindrome RNA). For 5′-OH and 3′-ovg hairpin dsRNA, an equimolar concentration of protein and fluorescent dsRNA (45 nM each for 5′-OH and 3′-ovg hairpin dsRNA) was used. The change in fluorescence intensity was recorded as a function of time, and the data were fit to a two-exponential equation [${A_1}{e^{ - {k_1}t}} + {A_2}{e^{ - {k_2}t}} + c$] to estimate the off-rates.

The on-rates (*k*_on_) were also measured at 25°C using the stopped-flow instrument. A fixed concentration of fluorophore-labeled 5′-OH and 3′-ovg hairpin dsRNA (10 nM) from syringe A was rapidly mixed with LGP2 (30–100 nM) from syringe B. The fluorescence intensity was recorded as a function of time and fitted to a three-exponential equation [${A_1}{e^{ - {k_1}t}} + {A_2}{e^{ - {k_2}t}} + {A_3}{e^{ - {k_3}t}} + c$] to obtain the observed rates of binding. The estimated rates (*k*_1_) with the two RNAs were line fit to the data to estimate the on-rates.

### ATP hydrolysis activity

The ATPase reactions were conducted using a constant concentration of LGP2 (10 nM) and increasing RNA concentrations (1–128 nM) in the presence of 1 mM ATP spiked with [γ-^32^P] ATP. The ATPase reaction was allowed to proceed for 60 and 120 minutes at 25°C in Buffer A. At each time point, the reactions were stopped by adding 8 M formic acid. The reaction mixtures were then spotted onto a PEI-Cellulose-F TLC plate (Merck) and developed in a 0.4 M potassium phosphate solution (pH 3.4). The TLC plates were exposed to a phosphorimager plate, and the radioactive signals were visualized using a Typhoon phosphor-imager. The extent of ATP hydrolysis was quantified by analyzing the counts from the resolved ATP and inorganic phosphate spots using the ImageQuant software.

### Biotin-streptavidin displacement assays to measure LGP2 translocation on dsRNA

The 27 bp dsRNAs labeled with 3′-DY547 (25 nM) and carrying biotin at the 5′ end, along with the desired 5′ modification at the opposite end, were incubated with monovalent streptavidin (60 nM, New England Biolabs) in Buffer A. Protein (25 nM) was added to the reaction mixture. The reactions were initiated by mixing this complex with ATP (2 mM) and free biotin (50 μM) as a chase to prevent the rebinding of streptavidin to the RNA. After the desired reaction time, the reactions were quenched with 10 mM EDTA and 0.5% SDS. The samples were loaded onto a 4–20% TBE gel (Invitrogen) and electrophoresed at 4°C. Subsequently, the gels were scanned using a Typhoon FLA 9500 laser-based scanner (GE Healthsciences). The fraction of streptavidin displaced from the biotinylated RNAs was quantitated using the ImageQuant TL software.

### Stopped-flow assay to measure LGP2 translocation activity

A 27-base pair dsRNA containing an internal Cy3-labeled nucleotide at the 24^th^ position from the 5′ppp end was synthesized to assess LGP2’s translocation ability on the dsRNA. The measurements were conducted using a stopped-flow instrument (Auto-SF 120, Kintek Corp, Austin, TX). A mixture of the internal Cy3 labeled dsRNA (40 nM) and LGP2 (40 nM) in Buffer A (pre-incubated at 25°C for 15 min) from syringe A was mixed with ATP (2 mM) and a 10-fold excess of RNA trap (unlabeled 5′ppp ds28 RNA) from syringe B. The fluorescence emission was measured using a 570 nm band-pass filter after excitation at 547 nm as a function of time. Data were globally fitted using the Kintek Explorer software.

## Results

### Human LGP2 is a monomer in solution and dimerizes on dsRNA

Human LGP2 protein was expressed in *E. coli* as an Ulp1 protease cleavable His-tag-SUMO fusion protein and in Sf9 insect cells as a TEV protease cleavable His-tag protein. The tag-less proteins were purified from both sources following the protocol used for the RIG-I protein (24,29,32). Although we obtained pure LGP2 protein from *E. coli*-expressed cells, the protein yield was low, prompting us to develop the insect cell expression system, which provided higher amounts of protein, but the yields of LGP2 from both sources were moderate. Most of the experiments in this study were carried out with human LGP2 purified from the insect cell-expression system. Some experiments, such as the ATPase and RNA translocation studies, were conducted with the *E. coli* expressed protein.

**Figure 2.**
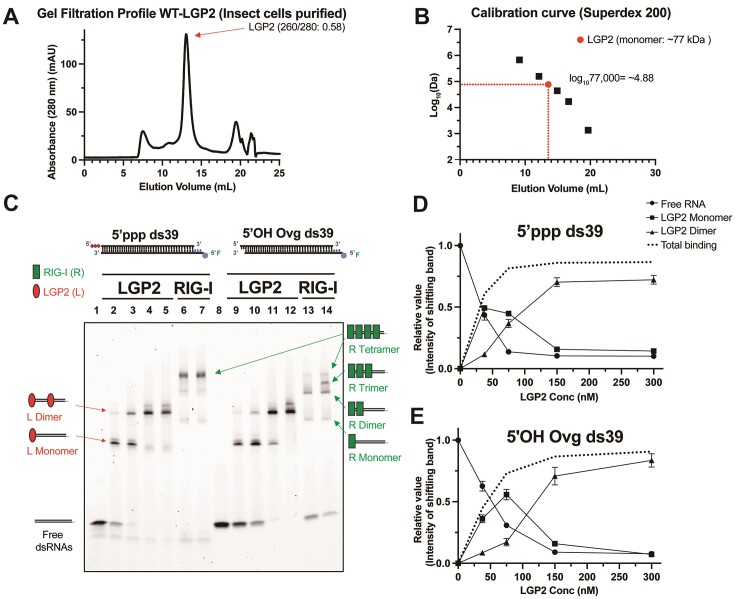
Assessment of LGP2 oligomerization with and without RNA. (**A**) Insect-cell expressed human LGP2 was subjected to gel-filtration analysis on Superdex 200 Increase 10/300 GL Cytiva column. The elution of LGP2 was monitored by 280 and 260 nm absorbances. (**B**) Superdex 200 calibration curve with standard proteins indicates that LGP2 elutes from the gel-filtration column with the expected molecular mass of a monomer. (**C**) A representative EMSA of LGP2 and RIG-I on 4–20% TBE gel shows protein multimerization on 5′ppp and 5′-overhang ds39 RNA (5′OH Ovg) in 2 mM ATP. Lanes 1 and 8: free RNA (25 nM); lanes 2–5 and 9–12: constant RNA titrated with different LGP2 concentrations (37.5, 75, 150, 300 nM); and lanes 6–7, 13–14 show constant RNA titrated with different RIG-I concentration (150- 300 nM). (**D**, **E**) LGP2 monomer and dimer species from lanes 2–5 and 9–12 in panel C were quantified and plotted against LGP2 concentration. The mean values and error bars of three replicates are shown.

A previous study reported that human LGP2 is a mixture of monomer and dimer in solution (33). To investigate the oligomeric state of LGP2, we analyzed the insect and *E. coli*-expressed LGP2 proteins employing gel-filtration chromatography. A comparison of the elution profile of LGP2 to the standard proteins shows that LGP2 purified from both sources is a monomer in solution (Figure 2A, B, Supplemental Figure S1A–C).

To investigate whether LGP2 can multimerize on RNA, we carried out EMSA on a ds39 RNA with a blunt-end containing a 5′-ppp or a 2-nt 5′OH-overhang. Both RIG-I and LGP2 were included in the study. To confine RIG-I/LGP2 binding to the blunt-end/5′-overhang end, we incorporated a DNA overhang at the distal end (Figure 2C). Additionally, we carried out a second set of EMSA experiments, adding a bulky streptavidin adduct to the DNA overhang to deter LGP2 binding to that end (Supplemental Figure S1D, E). The RNA footprint of LGP2 and RIG-I is about 9–10 base pairs (6,33); thus, depending on the protein-to-RNA ratio, we anticipated binding of 3–4 LGP2 or RIG-I molecules to the ds39 RNA.

At a concentration of 25 nM RNA and 150 or 300 nM RIG-I, we primarily observed RIG-I tetramers on the blunt-end 5′ppp ds39 RNA in the presence of ATP (Figure 2C). On the 5′-overhang RNA, we observed a distribution of RIG-I dimers and trimers, with almost no tetramers detected. These observations align with the previously reported strong binding and oligomerization of RIG-I on 5′ppp RNA and weaker affinity for the 5′-overhang RNA end (32).

In contrast to RIG-I, LGP2 only formed monomers and dimers on the 5′ppp ds39 RNA, even when the LGP2 concentration was increased to 300 nM protein (12 times the RNA concentration) (Figure 2C). No indication of further multimerization to trimers or higher-order oligomers was observed. The LGP2 monomer and dimer bands exhibited a minor lower mobility species, which could correspond to different conformations of the LGP2:RNA complex, such as open versus closed observed in the structural analysis of chicken LGP2 (33), leading to varying mobilities on EMSA. LGP2 was also bound to the 5′-overhang dsRNA as monomers and dimers, but quantitation indicated that slightly higher concentrations of LGP2 were required for binding and dimerization on the overhang RNA than the blunt-end RNA (Figure 2D, E). Therefore, the binding and dimerization of LGP2 on the 5′ppp blunt-ended ds39 RNA is slightly more efficient than the 5′-overhang ds39 RNA.

LGP2 monomers and dimers were also observed on the ds39 conjugated with streptavidin to one RNA end (Supplemental Figure S1D, E). This confirms that the dimer is not the result of two LGP2 molecules binding to each end of the RNA.

In conclusion, our findings demonstrate that LGP2 exists as a monomer in the absence of RNA, and it has the capability to form dimers on sufficiently long dsRNA. Interestingly, LGP2 does not undergo further multimerization into higher-order oligomers, as observed with RIG-I and published for MDA5 (5,30,34). This unique property of LGP2 may be important in modulating the RNA sensing mechanisms of RIG-I and MDA5. The implications are elaborated in the discussion section.

### RNA end binding properties of LGP2 compared to RIG-I

The results obtained from the EMSA experiments using the ds39 RNAs revealed that LGP2 exhibited a slightly stronger binding to the blunt-ended RNA, suggesting a potential preference of LGP2 for binding to specific RNA ends. Previous studies on chicken LGP2 and the C-terminal domain (CTD) of human LGP2 have hinted at an RNA-end binding preference (33,35); however, this aspect has not been quantitatively explored.

**Figure 3.**
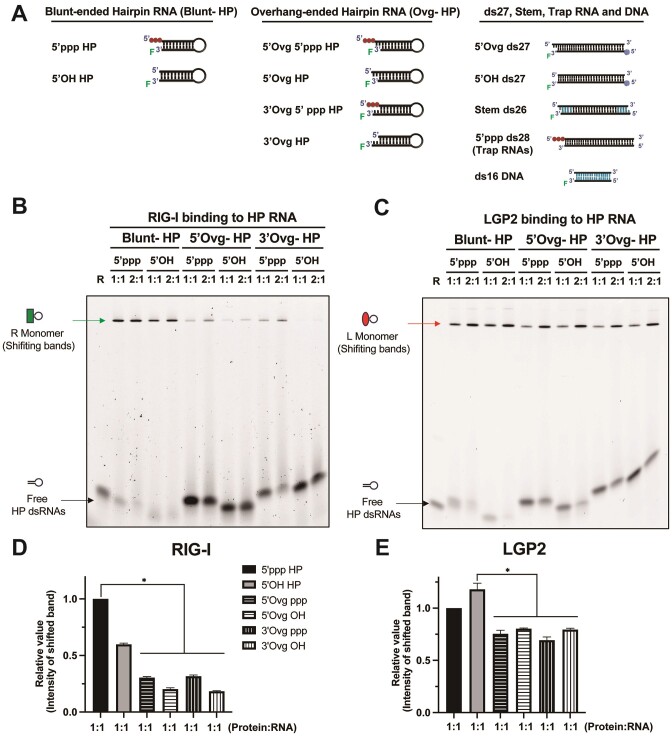
EMSA profile of RIG-I and LGP2 on the 10 bp hairpin RNAs. (**A**) Schematic representation of the hairpin RNA (HP RNA), 27 bp RNA (ds27), 26 bp stem RNA (Stem ds26), 28 bp palindrome RNA (5′ppp ds28) and 16 bp DNA (ds16 DNA) with different end modifications used in this study. Black-filled bp represents RNA segments, and blue-filled bp represents DNA segments. (B, C) Representative EMSA shows the binding of HP RNA with different end modifications (20 nM) to RIG-I (**B**) and LGP2 (**C**) (1:1 represents 20 nM RNA and protein, and 1:2 represents 20 nM RNA and 40 nM protein). The complexes were made in buffer A with 100 mM NaCl. R represents RIG-I and L is LGP2. (**D**, **E**) Quantification of the EMSA under 1:1 RNA:protein conditions for RIG-I **(B)** and LGP2 (C). The mean values and error bars of three replicates are shown. * *P* < 0.05. Error bars indicate SEM.

To further investigate the RNA-end binding property of LGP2 and avoid the interference of LGP2 dimerization, we synthesized a set of 10 base pair stem-loop hairpin RNAs (HP RNAs) capable of binding only one LGP2 molecule. These HP RNAs had either blunt ends with 5′OH or 5′ppp or 2-nucleotide 3′- and 5′-overhangs with 5′OH or 5′ppp (Figure 3A). RIG-I and LGP2 were incubated with the HP RNAs in 1:1 and 1:2 RNA:protein ratios, and the resulting complexes were analyzed using EMSA. Both formed a single shifted band on the gel, indicating the formation of a 1:1 complex (Figure 3B, C), consistent with their known structural footprint of 9–10 base pairs (6,33). As expected, RIG-I displayed a strong binding preference for blunt-end RNAs and formed weaker complexes on the overhang RNAs (32,36) (Figure 3B, D). Interestingly, LGP2 also exhibited a statistically better complex formation on the blunt-end RNAs compared to the overhang RNAs (Figure 3C, E).

These studies suggest that LGP2 has an RNA end preference. Because EMSA is a non-equilibrium assay and does not provide accurate *K*_D_ (dissociation constant) values for RNA binding, we employed fluorescence polarization titrations to quantify the *K*_D_ values of the LGP2:RNA complexes.

### LGP2 binds blunt-end RNAs with a higher affinity than overhang RNAs

**Figure 4.**
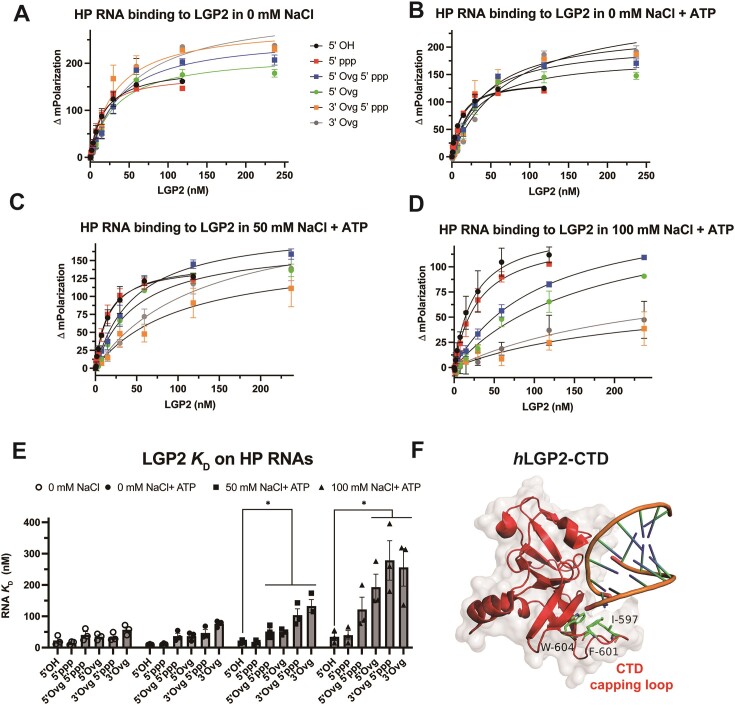
LGP2 shows a preference for binding to blunt-ended dsRNA. (**A–D**) Fluorescence polarization titrations of LGP2 binding to HP RNA with various end modifications. (**A**) No ATP, (B–D) 400 mM ATP in no salt (**B**), 50 mM NaCl (**C**) and 100 mM NaCl (**D**) in buffer A. (**E**) The equilibrium dissociation constant (*K*_D_) of LGP2 for the HP RNA (A–D) was assessed by fitting the fluorescence polarization titration curves and presented in bar chart form. The mean values and error bars of three replicates are shown. * *P* < 0.05. Error bars indicate SEM. (**F**) The CTD domain of human LGP2 (hLGP2-CTD) interacting with 8-bp 5′OH blunt-ended dsRNA (PDB: 3EQT). The three hydrophobic amino acids in the capping loop stack against the blunt end bp.

Fluorescence polarization-based titrations determined the *K*_D_ values of LGP2 under equilibrium conditions. In performing these experiments, we systematically tested the effect of RNA end and NaCl concentration on the *K*_D_ values to investigate any differential effects of salt on the affinities of blunt-end versus overhang RNAs. Increasing amounts of LGP2 protein were added to a constant concentration of fluorescein-labeled HP RNA. The fluorescence polarization of the HP RNA exhibited a hyperbolic increase with increasing LGP2 concentration (Figure 4A–D), and the dependency fitted a 1:1 stoichiometry binding model (Materials and Methods), enabling accurate estimation of the RNA *K*_D_ values (Figure 4E, Supplemental Table S1).

For the blunt-ended HP RNAs (5′OH and 5′ppp) in a buffer with 5 mM MgCl_2_ without added NaCl and ATP, LGP2 displayed a *K*_D_ value of approximately ∼10 nM. Under similar conditions, the overhang RNAs bound with a 3–7-fold weaker affinity than the blunt-end RNAs. Moreover, the 3′-overhang RNAs bound more weakly than the 5′-overhang RNAs (Figure 4E, Supplemental Table S1). The presence of ATP had minimal effect on the *K*_D_ values of both blunt-end and overhang RNAs (Figure 4A–B, E, Supplemental Table S1). This contrasts with a previous report that suggested that ATP enhances the binding of LGP2 to RNA (10).

Adding NaCl at concentrations of 50 mM and 100 mM decreased the binding affinity of LGP2 for the blunt-ended RNAs by about 2-fold and the overhang RNAs between 3- and 7-fold (Figure 4C, D, Supplemental Table S1). The decrease was more severe on the 3′-overhang RNAs than on the 5′-overhang RNAs. Thus, at 100 mM NaCl, the *K*_D_ values of LGP2 for the overhang RNAs were 4–10 times greater than those for the blunt-ended RNAs (Supplemental Table S1).

Adding NaCl also decreased the polarization amplitudes of the overhang RNAs selectively, especially that of the 3′ overhang RNA. In contrast, the polarization amplitudes of the blunt-ended RNAs were relatively unaffected by increasing NaCl concentration (Figure 4C, D, Supplemental Figure S2A–F). Assuming that NaCl does not affect the conformation of the LGP2-RNA complex. the polarization amplitude reflects the amount of the complex at equilibrium. Hence, adding salt decreases the amount of LGP2 complex on the overhang RNAs. Because the physiological salt is KCl, we also tested the effect of KCl on the binding of the blunt-end versus the overhang RNA. Similar to the NaCl, adding 50 mM and 100 mM KCl had little effect on the binding affinity and polarization amplitudes of the 5′OH blunt-ended RNA. However, the binding affinity and polarization amplitude of the 3′ overhang RNA decreased by 2–3-fold. The difference in the binding affinity of the blunt-end RNA versus 3′ overhang RNA at 100 mM KCl was about 10-fold (Supplemental Figure S4).

To validate the blunt-ended RNA-end binding preference of LGP2 on longer dsRNA, fluorescence polarization titrations were performed using ds27 RNA (27-bp dsRNA shown in Figure 3A) with either a blunt end or a 5′-overhang (Supplemental Figure S3). Like the HP RNAs, LGP2 exhibited stronger binding to the blunt-ended ds27 RNA than the overhang RNA. Furthermore, consistent with the HP RNAs, the binding of LGP2 to blunt-ended ds27 was less affected by increasing NaCl concentration, while the binding amplitudes of the 5′-overhang RNA decreased with higher salt concentrations (Supplemental Figure S3, Supplemental Table S1).

Based on these results, we conclude that LGP2 prefers binding to blunt-ended dsRNAs, and blunt-end binding is more salt-resistant than overhang RNA binding. This is consistent with the structural studies of LGP2, showing the capping loop of CTD interacting with the RNA blunt-end via several hydrophobic amino acids (Figure 4F) (33,35). Our studies also show that with overhang RNAs, LGP2 prefers the 5′-overhang vs. 3′-overhang, and unlike RIG-I, a 5′ppp at the blunt-end does not enhance LGP2 binding.

### LGP2 binds to Cap0 and Cap1 RNAs

**Figure 5.**
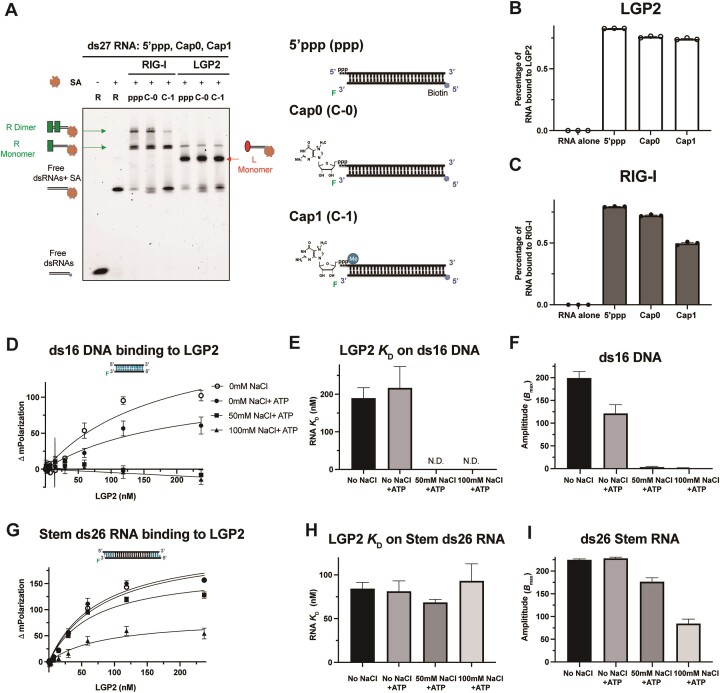
LGP2 binds to capped RNAs and shows weaker affinity to stem RNA. (**A**) Representative EMSA of RIG-I and LGP2 (60 nM) with 5′ppp (ppp), Cap0 (C-0) and Cap1 (C-1) ds27 RNAs (20 nM), SA represents monovalent streptavidin. (B, C) Quantification of the EMSA gel lanes from panel A shown as the percentage of RNA bound to RIG-I (**B**) or LGP2 (**C**). The dots represent three replicates. (**D**) Fluorescence polarization titrations measure the binding of LGP2 to ds16 DNA with or without 400 mM of ATP in no salt, 50 mM NaCl and 100 mM NaCl added conditions. (E, F) The *K*_D_ values of LGP2 binding to ds16 DNA (**E**) and the fluorescence polarization amplitudes (*B*_max_) (**F**) from data in panel (D) are presented. N.D. is non-detectable. The mean values and error bars of three replicates are shown. (**G**) Fluorescence polarization titrations measure the binding of LGP2 to stem ds26 RNA with or without 400 mM of ATP in no salt, 50 mM NaCl, and 100 mM NaCl added conditions. (H, I) The *K*_D_ values of LGP2 binding to stem RNA (**H**) and the fluorescence polarization amplitudes (*B*_max_) (**I**) from data in panel (**G**) are presented. The mean values and error bars of three replicates are shown.

In previous studies, it was demonstrated that RIG-I tolerates bulky adducts like the 7-methylguanosine (m7G) cap at the 5′ppp end (Cap0 dsRNA) (29) and also metabolite caps (30). However, ribose 2′-*O*-methylation on the first base from the 5′-end in the Cap1 RNA weakened RIG-I binding. Cytoplasmic mRNAs contain Cap1 modification, which helps to evade RIG-I recognition and aberrant immune responses from self-RNAs (31). To investigate whether LGP2 can recognize dsRNA ends with these modifications, we used EMSA and monitored complexes on 5′ppp, Cap0, and Cap1 ds27 RNAs (Figure 5A). The Cap0 ds27 contained an m7G group in the 5′-strand at the end of 5′ppp, and Cap1 contained an additional 2′-O-methylation in the ribose of the first base. We introduced a bulky biotin-streptavidin conjugate to hide the other blunt end in ds27 from LGP2/RIG-I recognition. We introduced a bulky biotin-streptavidin conjugate to hide the other blunt end in ds27 from LGP2/RIG-I recognition.

RIG-I showed almost complete binding to 5′ppp and Cap0 ds27, forming both monomer and dimer species, and 50% binding to Cap1 ds27 mainly as a monomer (Figure 5A, B). These results indicate that RIG-I’s binding is influenced by ribose 2′-O-methylation modification. In contrast, LGP2 bound to these RNAs mainly as a monomer and demonstrated nearly equal efficiency in forming complexes with 5′ppp, Cap0, and Cap1 ds27 RNAs (Figure 5A, C).

These results indicate that LGP2 tolerates both the m7G cap and the ribose methylation in the first nucleotide from the 5′-end, distinguishing it from RIG-I.

### LGP2 binds weakly to stem RNA compared to blunt-end RNA

RIG-I has a very low affinity for stem or internal dsRNA regions with estimated *K*_D_ values close to 1.2 mM with ATP (32,36). To assess the affinity of LGP2 for internal dsRNA regions, we employed a stem RNA mimic, where we introduced 4 bp dsDNA ends with a 1-nt overhang flanking a 17 bp dsRNA region (Figure 3A). By excluding the possibility of LGP2 binding to DNA (Figure 5D, E), any observed binding with the stem RNA mimic would be attributed to LGP2 binding to the internal dsRNA region. We examined the DNA binding activity of LGP2 using fluorescence polarization titration with a 16-bp dsDNA. At 0 mM NaCl, LGP2 bound to DNA with a *K*_D_ of approximately 200 nM, and the addition of ATP had little effect on the fitted *K*_D,_ but it lowered the amplitude of fluorescence polarization (Figure 5D, E, Supplemental Table S1). When we added salt to 50 mM and 100 mM, no discernible DNA binding was observed (Figure 5D-F, Supplemental Table S1). This finding is consistent with LGP2’s preference for binding an A-form backbone of the dsRNA (33).

Having established that LGP2 does not bind DNA at 50 mM and 100 mM NaCl, we conducted fluorescence polarization titrations with the stem RNA. At 0 mM NaCl, LGP2 bound to the stem RNA with a *K*_D_ of approximately 80 nM, which is approximately 8 times weaker than the affinity for blunt-ended RNA. Adding ATP did not affect LGP2’s affinity for the stem RNA (Figure 5G, H). However, increasing NaCl to 50 and 100 mM decreased the polarization amplitudes (Figure 5I), mirroring the observations made with the overhang RNAs.

Thus, LGP2 binds to internal regions of dsRNA but with an affinity 5–8 times weaker than that for the blunt end. Compared to RIG-I (36), however, LGP2 has a much higher affinity (>500 fold) for internal dsRNA regions. Thus, under similar conditions, LGP2 is expected to outcompete RIG-I in binding to internal regions in dsRNA.

### The kinetic basis for blunt-end RNA binding of LGP2

We wondered if LGP2’s preference for blunt-end RNA is due to its rapid rate of RNA binding or a slower rate of dissociation. The affinity of LGP2 for RNA, quantified by the equilibrium *K*_D_ value, depends upon the on-rate (*k*_on_) and off-rate (*k*_off_) of the LGP2:RNA complex (*K*_D_ = *k*_off_/*k*_on_). The higher affinity of LGP2 for blunt-end RNA could stem from a larger *k*_on_ value or a smaller *k*_off_ value (longer complex lifetime on the RNA). To understand the kinetic basis for blunt-end RNA preference, we conducted a comparative analysis of the RNA association and dissociation kinetics between the blunt-end RNA and the 3′-overhang RNA.

**Figure 6.**
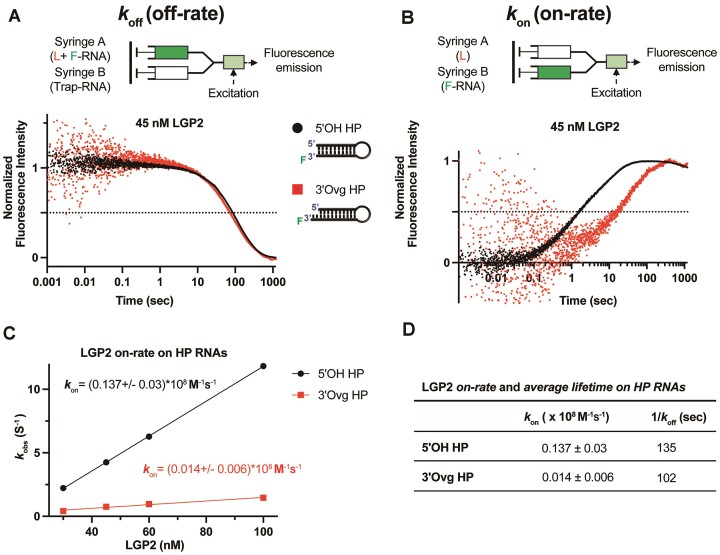
Off and on-rate of 5′OH and 3′Ovg HP RNAs from LGP2. (**A**) Dissociation kinetics of LGP2 from HP RNAs. A preformed complex of LGP2 (45 nM) and fluorescein-labeled RNA (5′OH or 3′Ovg HP, 45 nM) was chased with a 10-fold excess of unlabeled trap-RNAs (5′ppp ds28 palindrome RNA) in a stopped-flow instrument at 25°C. The time-dependent decrease in fluorescence intensity was fit to a sum of two exponentials to assess the off-rates (Supplemental Table S2). (**B**) Association kinetics of LGP2 to HP RNAs. A solution of LGP2 (45 nM) was rapidly mixed with 5′OH or 3′Ovg HP (10 nM) at 25°C. The time-dependent increase in fluorescence intensity upon binding of LGP2 to RNA was fit to a sum of three exponentials to obtain the binding rates (Supplemental Table S3). (**C**) The observed rate of binding (*k*_obs_) is plotted against increasing LGP2 (30 to 100 nM) for the 5′OH or 3′Ovg HP RNAs. The slope of the line provides the on-rate constant (*k*_on_). (**D**) The on-rate constant (*k*_on_) and average lifetime (1/*k*_off_) of LGP2 are tabulated.

To measure the *k*_off_ values, LGP2 was incubated with fluorescein-labeled HP RNA and chased with unlabeled RNA (Figure 6A). The fluorescein-labeled HP RNA has a higher fluorescence when bound to LGP2 than the free RNA in the solution. Therefore, the dissociation of LGP2 from the labeled RNA upon adding the chase led to a time-dependent decrease in fluorescence intensity. The RNA dissociation kinetics showed two phases and fit a sum of two exponentials (Figure 6A, Supplemental Figure S5A, B, Supplemental Table S2), suggesting either two populations of LGP2:RNA complex dissociating at different rates or a multistep dissociation mechanism. To simplify the interpretation, we calculated the amplitude-weighted lifetime of the LGP2:RNA complex on the two RNAs (Materials and Methods). This analysis indicated that the average lifetimes (inversely proportional to the off-rates) of LGP2 on the 5′OH blunt-ended RNA and the 3′-overhang RNA were approximately 135 s and 102 s, respectively (Figure 6D). These values, although different, do not account for the ∼10-fold difference in *K*_D_ values observed for the two RNA types.

To determine the *k*_on_ values, we mixed LGP2 with the fluorescent RNA rapidly and monitored the increase in fluorescence intensity, reflecting complex formation between LGP2 and RNA over a time range of 2 ms to 1000 s (Figure 6B). The binding kinetics were measured at increasing concentrations of LGP2 to determine the bimolecular *k*_on_ (Supplemental Figure S5C–J). An overlay of the binding kinetics shows faster binding of LGP2 to the 5′OH blunt-ended RNA than the 3′-overhang RNA. The fluorescence amplitude with the blunt-ended RNA was greater than the 3′-overhang HP RNA; hence, the data were normalized for comparison (Figure 6B). The binding kinetics exhibited multiple phases and were fitted with a sum of exponentials (Supplemental Table S3). The multiple phases indicate a complex RNA binding mechanism with an initial RNA binding step followed by conformational changes. Each rate constant was plotted against LGP2 concentration. Only the rate of the fast phase showed a linear increase with increasing LGP2 concentration (Figure 6C, Supplemental Figure S5K, L); hence, we used this phase and the slope to determine the bimolecular *k*_on_ values for the initial complex formation.

The *k*_on_ value of the 5′OH blunt-ended HP RNA (1.4 × 10^7^ M^−1^s^−1^) is 10-fold greater than the 3′-overhang HP RNA (1.4 × 10^6^ M^−1^s^−1^) (Figure 6C, D). Thus, the around 10-fold difference in *K*_D_ values of the two RNAs is due to the difference in the *k*_on_ rather than the *k*_off_. The rate measurements of LGP2 with the blunt-end RNA are consistent with previously published values obtained from single-molecule experiments (10).

The kinetic analysis reveals that LGP2’s higher affinity for the blunt-end RNA results from its fast binding rate to this particular RNA. Previous studies (35) have shown that LGP2’s C-terminal domain (CTD) has a higher affinity to blunt-end RNA while not showing the same affinity for overhang RNA. Consequently, the different on-rates and the distinction in binding mechanisms for the two RNA ends can be attributed to LGP2’s CTD, which may influence how LGP2 regulates RIG-I/MDA5.

### LGP2 hydrolyzes ATP at a 5-10-fold slower rate than RIG-I

**Figure 7.**
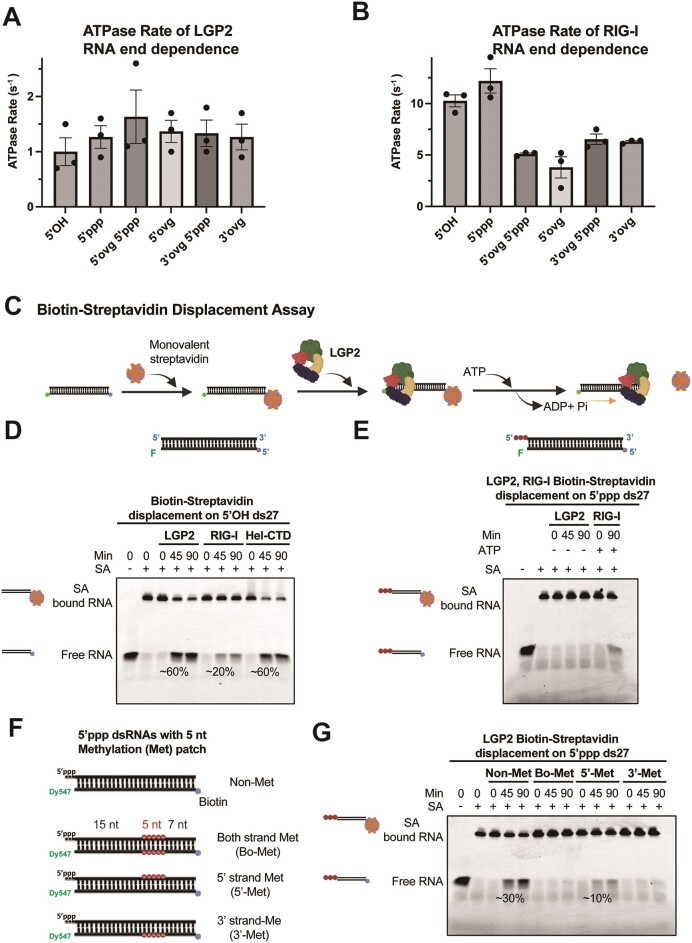
ATP hydrolysis and LGP2 translocation on dsRNA. (A, B) The bar chart shows the ATP hydrolysis rate of LGP2 (**A**) and RIG-I (**B**) in the presence of HP RNA with various end modifications tabulated in Supplemental Table S4. (**C**) Biotin–streptavidin displacement assay schematic. (**D**) Time course of biotin–streptavidin displacement from 5′OH ds27 RNA (25 nM) by LGP2 (75 nM), RIG-I (75 nM) and RIG-I Hel-CTD (75 nM) with 2 mM ATP and free biotin (50 μM) as a chase to prevent the rebinding of streptavidin to the RNA. (**E**) Time course of biotin-streptavidin displacement from 5′ppp ds27 RNA (25 nM) by LGP2 (25 nM), RIG-I (25 nM) with or without 2 mM ATP and free biotin (50 μM). (**F**) 5′ppp ds27 RNAs with ribose methylation patch on one or both strands. (red dots, 5-nt patch of ribose 2′-*O*-methylated nucleotides placed 15 bp downstream of the 5′ppp end). (**G**) Time course of biotin-streptavidin displacement by LGP2 (25 nM) on the methylated patched 5′ppp dsRNAs (25 nM) with 2 mM ATP and free biotin (50 μM).

The ATP hydrolysis activity of LGP2 is important for activating MDA5 (10). To determine if the type of RNA end affects the ATPase function of LGP2, we measured the ATPase activity using the panel of HP RNAs with different RNA ends employed above in the RNA binding studies. In this study, we utilized *E. coli*-expressed LGP2, which has comparable RNA binding and ATPase functions as the insect cell-expressed LGP2 (Supplemental Figure S6A–D). In contrast to the previous study (10), neither protein showed a significant basal ATP hydrolysis rate without RNA (Supplemental Figure S6C). The amount of inorganic P_i_ produced by LGP2 in the absence of RNA was similar to the background level of P_i_ in the radiolabeled ATP stock (Supplemental Figure S6C). The ATPase activity on the panel of HP RNAs was measured at increasing RNA concentrations to determine the maximal ATPase rate under conditions where all the LGP2 molecules are bound to RNA. Under these conditions, LGP2 exhibited ATP hydrolysis at a rate of approximately 0.8 to 1 s^−1^, and this rate remained unaffected by the RNA end (Figure 7A, Supplemental Table S4). In similar conditions, RIG-I displayed ATP hydrolysis rates of around 10 s^−1^ on blunt-ended RNAs and approximately 5 s^−1^ on overhang RNAs (Figure 7B, Supplemental Table S4). These results indicate that LGP2 hydrolyzes ATP 5–10 times slower than RIG-I.

### The ATPase activity promotes LGP2 translocation on RNA

RIG-I uses its ATPase activity to translocate along RNA (32,37), and it has been demonstrated that translocation generates sufficient force to displace streptavidin bound to biotin at the end of a 27-bp RNA (32). To investigate whether LGP2 exhibits similar translocation and force generation capabilities as RIG-I, we conducted biotin-streptavidin displacement assays using blunt-ended 5′OH and 5′ppp ds27 RNAs (Figure 7C–E). The biotin was present at the RNA end distal from the blunt-end and conjugated with monovalent streptavidin. The translocation assays were performed with full-length RIG-I, RIG-I HEL-CTD and *E. coli-*expressed LGP2 in the presence of ATP and analyzed using EMSA. In the native gel, the free RNA migrates more slowly when bound to streptavidin, and the loss of this gel shift indicates the displacement of streptavidin by the translocating helicase. The experiments were carried out with 25 nM RNA and 75 nM protein. Within 45 min, LGP2 displaced approximately 60% of the streptavidin, while full-length RIG-I displaced only 20% (Figure 7D). Because RIG-I and LGP2 bind to the ds27 RNA comparably (Figure 5A), we argue that the greater percentage of streptavidin displaced is due to greater force production by LGP2 than RIG-I. Interestingly, the RIG-I HEL-CTD construct, lacking the linker and CTD; thus, structurally similar to LGP2, displaced 60% of the streptavidin, similar to LGP2. Notably, the streptavidin displacement activity was absent when ATP was removed, highlighting the dependence on ATP for force production (Figure 7E).

To investigate any strand-specificity of the translocation activity, we introduced a 5-bp ribose-methylation patch 15 bp downstream of the blunt end in the 5′ppp blunt-ended ds27 (Figure 7F). These sets of RNAs were used in our previous translocation studies with RIG-I (32). The 15 bp region allows LGP2 to bind the RNA end, but if methylation hinders its translocation ability, the streptavidin will remain intact. Three types of ds27 RNAs were generated: one with the methylation patch on both strands, another with the patch on the 5′-strand, and a third with the patch on the 3′-strand. The experiments were carried out with 25 nM RNA and 25 nM LGP2. The unmethylated 5′ppp ds27 showed 30% streptavidin displacement, whereas both strands containing the methylation patch resulted in no displacement (Figure 7G). The 30% displacement efficiency on the unmethylated 5′ppp RNA is lower than the 60% observed with the 5′OH RNA. This could be due to the lower LGP2 concentration in the 5′ppp RNA experiments or the specific end modification. No displacement was observed when the methylation patch was present on the 3′-strand, but approximately 10% occurred when the patch was on the 5′-strand. These findings align with the translocation activity of RIG-I (32) and indicate that LGP2 utilizes its interactions with the 3′-strand to displace the streptavidin from the RNA.

We also used a real-time PIFE (protein-induced fluorescence enhancement) assay reported in previous studies of RIG-I (32) to confirm the translocation activity of LGP2. In this assay, we labeled the ds27 RNA with a Cy3 fluorophore at the 24th position from the 5′ppp blunt-end. An increase and decrease in fluorescence intensity after mixing LGP2 with the RNA provided evidence for LGP2 translocation (Supplemental Figure S6E). As LGP2 arrives close to Cy3, the PIFE is detected and maximizes as LGP2 goes over the Cy3 fluorophore. Subsequently, when LGP2 leaves Cy3, the PIFE decreases. The unlabeled RNA trap prevented the rebinding of LGP2 to the Cy3 labeled RNA.

In summary, our findings demonstrate that although LGP2 hydrolyzes ATP slower than RIG-I, it displaces a greater percentage of streptavidin than RIG-I. Thus, LGP2 can efficiently use its ATPase activity to power the dissociation of streptavidin.

Note that LGP2 at concentrations of 25 nM and 75 nM is bound to the ds27 RNA mainly as a monomer (Figure 5A). Hence, we show here that the monomeric LGP2 can displace streptavidin-biotin interactions. It will be interesting to determine the translocation activity of the LGP2 dimer.

## Discussion

In this study, we employed quantitative biochemical and biophysical methods to investigate protein oligomerization, RNA binding preference, and ATPase activity of human LGP2 protein. Our findings reveal that LGP2 predominantly exists as a monomer when in solution. However, when exposed to sufficiently long dsRNA, it demonstrates the ability to dimerize—a unique characteristic that distinguishes LGP2 from RIG-I and MDA5, both of which can form higher-order oligomers upon encountering dsRNA (30,34,38).

By measuring the RNA *K*_D_ values, we discovered that LGP2 exhibits a stronger affinity for blunt-ended RNA compared to RNA ends with overhangs or internal regions, like RIG-I. However, LGP2’s affinity for the overhang RNA ends and internal dsRNA regions is much greater than that of RIG-I. We show that LGP2 hydrolyzes ATP only in the presence of RNA, and the ATPase activity is equally stimulated by various dsRNAs, regardless of the type of RNA end. Furthermore, similar to RIG-I, LGP2 utilizes its ATPase activity to translocate and effectively displace streptavidin attached to biotin at the end of a ds27 RNA.

By elucidating the intricate details of LGP2’s RNA binding and translocation behavior, our study provides valuable insights into the basic properties of LGP2 and potential ways in which its unique characteristics could be involved in regulating the RNA-sensing steps of RIG-I and MDA5.

### Structural basis of LGP2’s preference for binding blunt-ended RNA end

The preference of LGP2 for binding blunt-ended RNA can be attributed to its C-terminal domain (CTD) (Figure 1B, C). Structural analyses of the isolated CTD (human) and full-length LGP2 (chicken) have revealed important details about the binding preferences of LGP2 (28,33,35). Specifically, several hydrophobic residues (I597, F601, W604 in human LGP2) in the capping loop of the CTD (spanning 592–604) stack against the terminal base pair of the dsRNA (Figure 4F). Additionally, the full-length chicken LGP2 structure shows that residues from the HEL1 loop interact with the 3′-end ribose of the blunt-end RNA. These interactions of the capping loop and HEL1 loop are altered due to the presence of a 3′-overhang (Supplemental Figure S7A, B).

The hydrophobic interactions with the blunt-end help explain why LGP2’s binding to blunt-end RNA is less sensitive to increasing salt concentration than its binding to overhang and internal dsRNA. While a structure with a 5′-overhang is not available, there seems to be more space to accommodate the 5′-overhang. This observation explains the higher affinity of LGP2 for 5′-overhang RNA compared to 3′-overhang RNA, as observed in our studies.

In the case of RIG-I, the CTD is the first to contact the RNA end and load the RNA stem into the helicase domain (32). The CTD is connected to Hel2 in all RIG-I-like receptors through a helical pincer structure. However, there are notable differences between LGP2 and RIG-I in the arrangement of the pincer and CTD. In LGP2, the pincer helix directly connects to the CTD, whereas in RIG-I, the CTD is flexibly linked to the pincer helix via a linker (6,33). The initial interactions between the flexible CTD in RIG-I and the RNA end are diffusion-limited, with a rate constant of 5 × 10^8^ M^−1^s^−1^ (32). On the other hand, the constrained CTD-pincer linkages in LGP2 result in an approximately 40-fold slower rate constant (1.4 × 10^7^ M^−1^s^−1^) of LGP2 binding to blunt-end RNA compared to RIG-I.

Although LGP2 does not exhibit a specific preference for binding to 5′ppp RNAs, the structure of chicken LGP2 revealed multiple interactions with the 5′ppp group (33). In blunt-ended RNA, the phosphates coordinate with an Mg ion and engage in specific interactions with residues from the Hel2 loop (H406, S407 and N408) and the capping loop (K634) (Supplemental Figure S7C). In human LGP2, the corresponding residues in the Hel2 loop are N408, S409, and S410, and in the capping loop is R636, which are not identical to the residues found in chicken LGP2 (Supplemental Figure S7D). It is unknown how the amino acid differences in human vs. chicken LGP2 affect the interactions with the 5′ppp. However, our studies indicate that the presence of the 5′ppp group does not enhance LGP2’s affinity for blunt-ended RNA.

### Implications of LGP2’s RNA binding specificities for the regulation of RIG-I and MDA5’s RNA sensing mechanism

LGP2 is crucial in modulating the RIG-I/MDA5 signaling pathway immune responses. Multiple studies have consistently shown that LGP2 inhibits RIG-I and activates MDA5 (1,3,13–16). However, LGP2 can also inhibit MDA5 at high concentrations by interfering with downstream ubiquitination steps (18,19). This concentration-dependent effect and feedback regulation by LGP2 is essential for preventing excessive immune responses that could be harmful.

Our study provides insights into LGP2’s interactions with dsRNAs, including 5′ppp blunt-ended dsRNA, which RIG-I recognizes as a pathogen-associated molecular pattern (PAMP). Depending on its concentration, LGP2 may competitively inhibit RIG-I’s viral RNA response through RNA competition and the aforementioned feedback inhibition mechanism. LGP2 exhibits a higher affinity for overhang RNAs and internal dsRNA regions than RIG-I; thus, LGP2 may prevent RIG-I from associating with self-RNAs and avoid undesirable immune responses. Previous models have proposed such mechanisms (11), but further investigations are necessary to determine the precise regulatory mechanism with the contribution of LGP2’s RNA competition relative to its inhibition of steps downstream steps of the RNA binding.

In contrast to its inhibitory role on RIG-I, the current model suggests that LGP2 nucleates MDA5 multimerization on long dsRNA (9,17). Comparing the RNA binding affinity of LGP2 determined in our study with published values for MDA5 (4), it becomes evident that LGP2 exhibits a much stronger affinity for dsRNA than MDA5. The RNA end binding specificity of LGP2 may play a critical role in specifically nucleating MDA5 multimers on blunt-ended viral RNA ends rather than self-derived dsRNAs. The rapid binding rate demonstrated by LGP2 in our study is also desirable for its role as a nucleating factor. The higher association rate of LGP2 for blunt-end RNA versus RNA with an overhang indicates that LGP2 will competitively identify viral RNA (with blunt-ends) over self RNAs (without such ends) and help in the preferential nucleation of MDA5 filament on viral RNAs.

An intriguing avenue of exploration lies in the examination of LGP2’s binding preferences to ADAR1 (adenosine deaminase acting on RNA)-modified over unmodified dsRNAs. A recent study unveiled LGP2’s pivotal role in activating MDA5 in ADAR1-deficient cells (39). ADAR1 editing converts the adenosine bases in RNA to inosine, resulting in a wobble I:U base pair. Interestingly, internal I:U base pairs are 2 to 2.4 kcal/mol less stable than the A:U base pair, but terminal I:U base pairs are 0.8 to 0.9 kcal/mol more stable than A:U (40). The internal I:U base pairs resulting from RNA editing downregulate MDA5-mediated immune responses from endogenous RNAs (41). It will be interesting to explore how A to I modifications affect the binding affinity of LGP2 to dsRNAs and whether they alter the MDA5 activation mechanism. A molecular understanding of the differences in LGP2 end-binding and internal RNA binding modes to edited and unedited RNAs will provide insights into the role of LGP2 in ADAR1-mediated downregulation of MDA5 activation.

### LGP2 recognizes capped RNAs

Viral RNAs are often capped and 2′-O methylated, weakening RIG-I’s affinity for the self RNAs and preventing an unwanted immune response (29,42,43). Our studies show that LGP2’s RNA binding affinity is not affected by m7G capping or 2′-O-methylation of the ribose at the 5′-end (Figure 5A–C). Previous studies have shown that H830 in RIG-I interacts with the 5′-end ribose 2′-OH and this interaction is disrupted by 2′-O-methylation (29). This histidine is conserved in LGP2 as well, but since the blunt end of the dsRNA is located 1 bp deeper in the RNA binding channel than in RIG-I, the H574 (chicken) and H576 (human) is not interacting with the 5′-end ribose 2′-OH of the first bp but within interaction distance with the ribose 2′-OH of the second bp (28,33,35). We predict that a Cap2 RNA containing methylation modification at both the first and second bp will negatively affect the binding of LGP2 to RNA. However, this remains to be tested.

### LGP2’s inability to form multimers on dsRNA and possible roles of a dimer

LGP2 has a high affinity for dsRNA and a footprint of approximately 10 bp on dsRNA; hence, we expected three or four LGP2 molecules to assemble on the ds39 (39-bp) RNA (Figure 2C). However, in our observations, even at high concentrations of LGP2 relative to RNA, only two molecules of LGP2 bound to the 5′ppp blunt-ended dsRNA, whereas four molecules of RIG-I were observed on the same RNA in the presence of ATP. This result is consistent with a prior study, which indicated that human LGP2 fails to establish filaments within extended dsRNA despite sufficient LGP2 for filament formation (17). Nonetheless, the dimerization tendency of LGP2 is intriguing.

A possible explanation for confining the oligomers to dimers is that two LGP2 molecules bind to dsRNA in a head-to-head configuration through specific protein-protein interactions mediated by the RNA. We used AlphaFold multimer to predict the interactions between two LGP2 molecules. Of the five possibilities generated by AlphaFold multimer, four were in the head-to-head, and one was in the head-to-tail configuration (Supplemental Figure S8A–G). These models need to be experimentally validated.

If LGP2 multimerization is confined to a dimer, this property could be advantageous as a nucleating factor for MDA5 multimerization. By avoiding the formation of multimers on long dsRNA, LGP2 will prevent competition with MDA5 multimerization. Moreover, LGP2 dimer in a head-to-head configuration would not be able to translocate efficiently as the two molecules would move towards each other; this property would prevent LGP2 from stripping MDA5 filaments off the dsRNA and may stabilize the filament. This characteristic of LGP2 can also be beneficial in reducing competition with RIG-I multimers on viral RNAs.

### Potential roles of the ATPase-driven translocation activity of LGP2

A previous study reported that LGP2 does not translocate as RIG-I does in single-molecule conditions (10). However, our studies show that LGP2 can translocate and displace high-affinity bonds between streptavidin and biotin. Thus LGP2, similar to helicases (44,45), can use ATP hydrolysis for force production. Although the exact mechanism of force production is unknown, structural studies have highlighted closed and semi-closed conformational states of chicken LGP2, whereby the ATP-bound state is closed, making tight interactions around dsRNA, and the nucleotide unbound state is more relaxed (33). These protein structural transitions coupled to ATP binding and hydrolysis can lead to unidirectional translocation and force generation.

RIG-I translocates on dsRNA with different ends; however, previous studies showed that due to throttling at the 5′ppp end, the translocation rate of RIG-I is faster on non-ppp RNAs than 5′ppp RNA (32). This preference would prevent RIG-I from forming stable complexes on non-ppp RNAs, which is advantageous for preventing unwanted immune responses from endogenous RNAs (46,47). Additionally, RIG-I employs ATPase-driven translocation to multimerize on 5′ppp blunt-ended dsRNA (32,47), potentially facilitating CARD-CARD interactions for interactions with the downstream adapter proteins.

The precise function of LGP2’s ATPase-driven translocation and protein displacement activity remains unclear. LGP2’s translocation is powered by the hydrolysis of ATP, as shown in Figure 7E. Interestingly, certain mutants of LGP2 that lack ATPase activity fail to activate MDA5 (10). This suggests that LGP2’s translocation and force generation activities likely play a crucial role in the activation of MDA5. It is plausible that LGP2’s translocation activity reorganizes long MDA5 multimers into shorter fragments, potentially enhancing the signaling process (9,17). In addition to nucleating the formation of MDA5 filaments, LGP2 may stabilize the filament by hindering MDA5 molecules from detaching from the RNA end during ATP hydrolysis (34).

Because LGP2 can displace streptavidin from biotin, it raises the possibility that it can also displace nucleoproteins that are masking viral RNAs. The displacement can make the viral RNAs inaccessible for RIG-I and MDA5 binding. The translocation activity may help LGP2 form specific complexes on viral RNAs by preventing its entrapment on endogenous dsRNAs. The discovery that LGP2 can translocate on dsRNA raises numerous questions that require further investigation through additional research.

## Supplementary Material

gkad1106_Supplemental_File

## Data Availability

The Data underlying this article are available in Mendeley Data at https://doi.org/10.17632/5thfckdhh2.1.
