# Supplementary material for: Unraveling blunt-end RNA binding and ATPase-driven translocation activities of the RIG-I family helicase LGP2
Source: Nucleic Acids Res. 2023 Nov 28;52(1):355–69. doi: 10.1093/nar/gkad1106 (PMC10783506; doi:10.1093/nar/gkad1106)
Supplement: gkad1106_Supplemental_File [file gkad1106_supplemental_file.pdf]

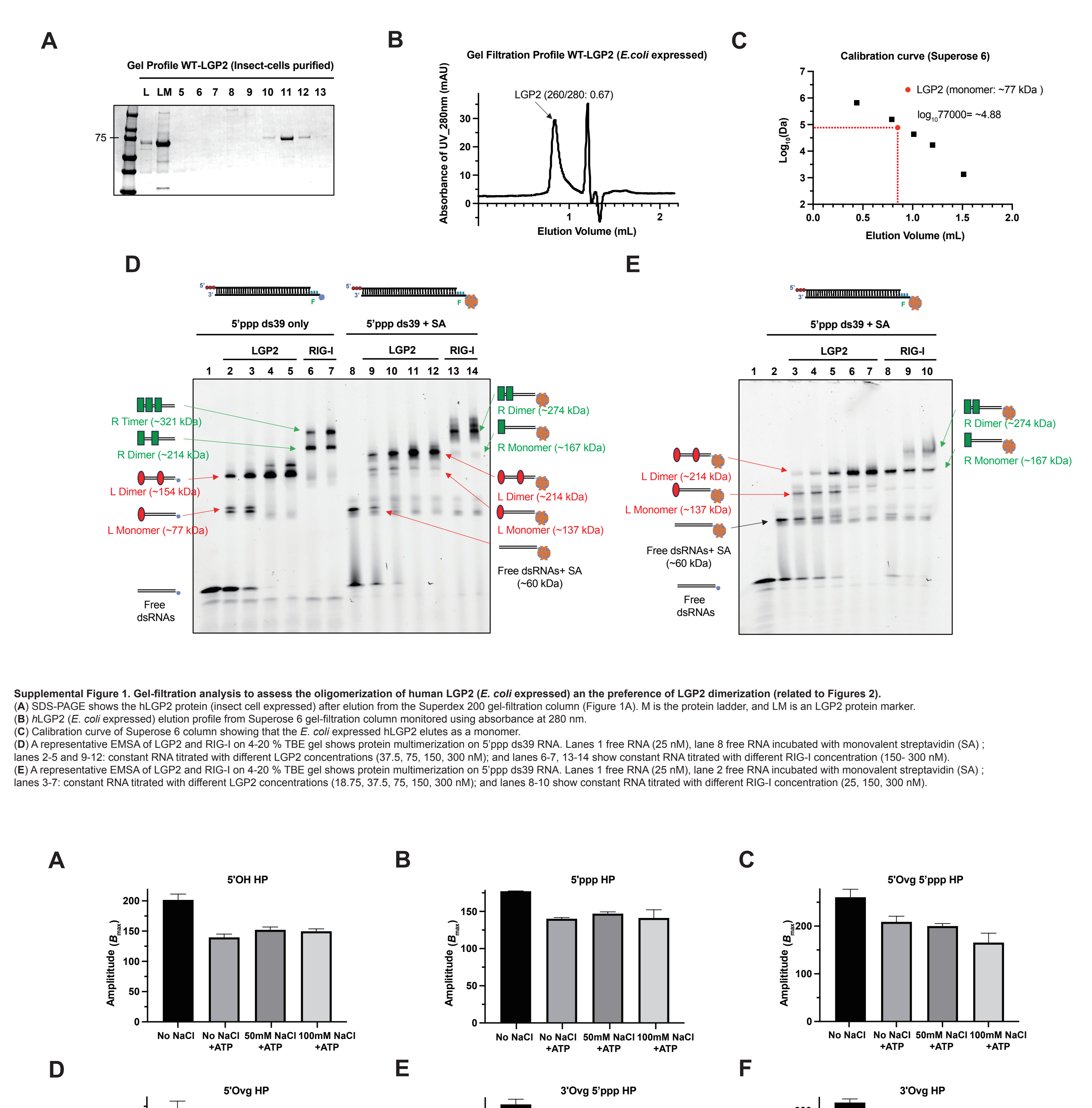

Supplemental Figure 1. Gel-filtration analysis to assess the oligomerization of human LGP2 (*E. coli* expressed) an the preference of LGP2 dimerization (related to Figures 2).
(**A**) SDS-PAGE shows the hLGP2 protein (insect cell expressed) after elution from the Superdex 200 gel-filtration column (Figure 1A). M is the protein ladder, and LM is an LGP2 protein marker.
(**B**) *h*LGP2 (*E. coli* expressed) elution profile from Superose 6 gel-filtration column monitored using absorbance at 280 nm.
(**C**) Calibration curve of Superose 6 column showing that the *E. coli* expressed hLGP2 elutes as a monomer.
(**D**) A representative EMSA of LGP2 and RIG-I on 4-20 % TBE gel shows protein multimerization on 5'ppp ds39 RNA. Lanes 1 free RNA (25 nM), lane 8 free RNA incubated with monovalent streptavidin (SA) ; lanes 2-5 and 9-12: constant RNA titrated with different LGP2 concentrations (37.5, 75, 150, 300 nM); and lanes 6-7, 13-14 show constant RNA titrated with different RIG-I concentration (150- 300 nM).
(**E**) A representative EMSA of LGP2 and RIG-I on 4-20 % TBE gel shows protein multimerization on 5'ppp ds39 RNA. Lanes 1 free RNA (25 nM), lane 2 free RNA incubated with monovalent streptavidin (SA) ; lanes 3-7: constant RNA titrated with different LGP2 concentrations (18.75, 37.5, 75, 150, 300 nM); and lanes 8-10 show constant RNA titrated with different RIG-I concentration (25, 150, 300 nM).

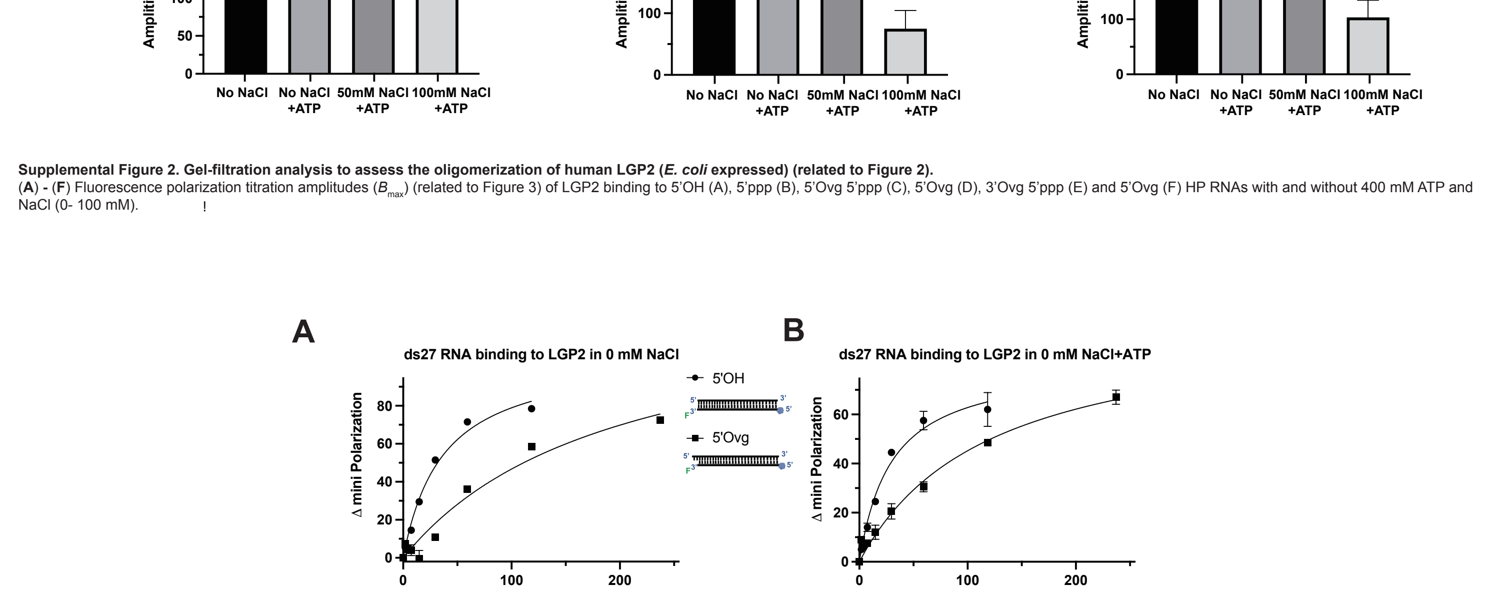

Supplemental Figure 2. Gel-filtration analysis to assess the oligomerization of human LGP2 (*E. coli* expressed) (related to Figure 2).
(**A**) - (**F**) Fluorescence polarization titration amplitudes (B_{max}) (related to Figure 3) of LGP2 binding to 5'OH (A), 5'ppp (B), 5'Ovg 5'ppp (C), 5'Ovg (D), 3'Ovg 5'ppp (E) and 5'Ovg (F) HP RNAs with and without 400 mM ATP and NaCl (0- 100 mM).

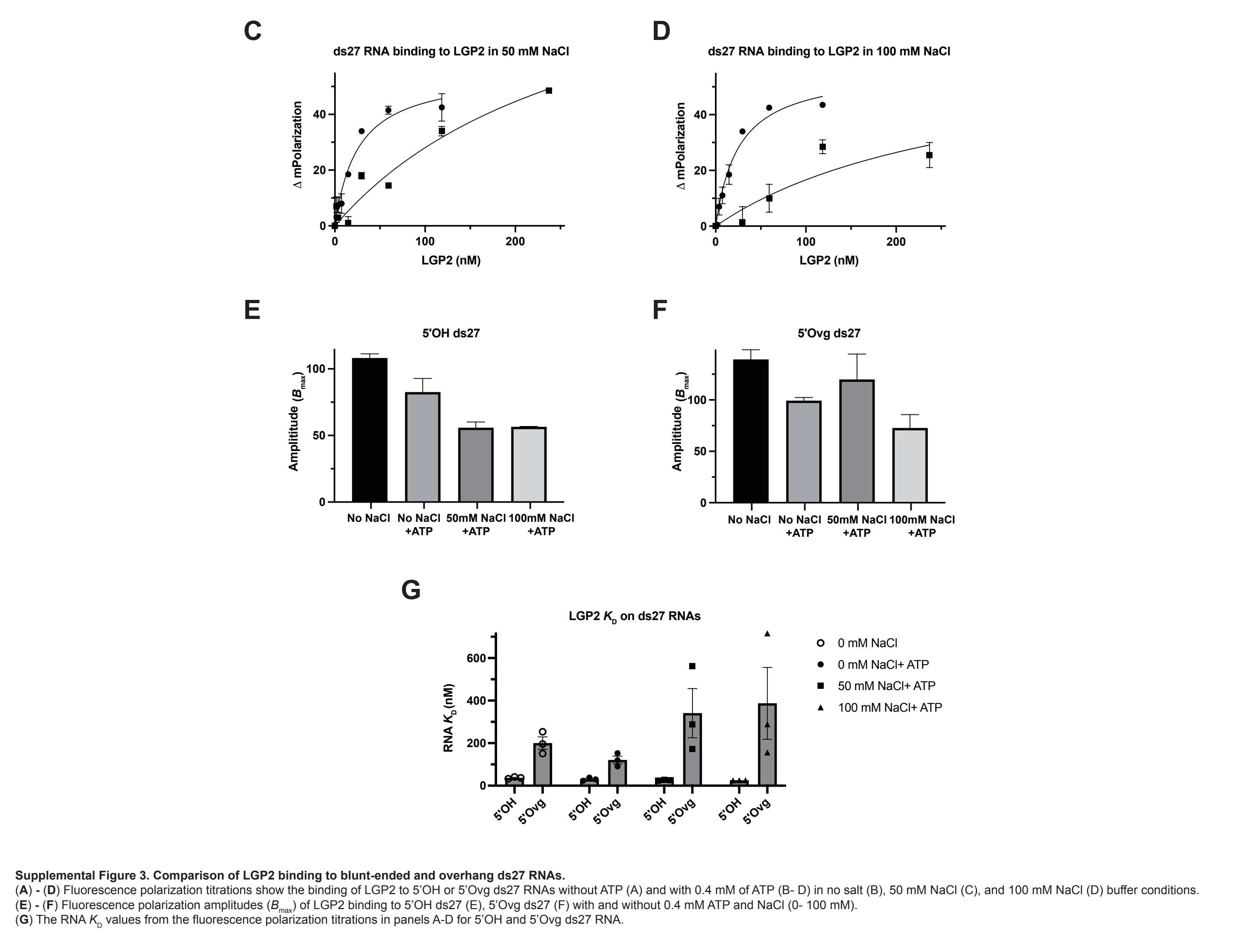

Supplemental Figure 3. Comparison of LGP2 binding to blunt-ended and overhang ds27 RNAs.
(**A**) - (**D**) Fluorescence polarization titrations show the binding of LGP2 to 5'OH or 5'Ovg ds27 RNAs without ATP (A) and with 0.4 mM of ATP (B- D) in no salt (B), 50 mM NaCl (C), and 100 mM NaCl (D) buffer conditions.
(**E**) - (**F**) Fluorescence polarization amplitudes (B_{max}) of LGP2 binding to 5'OH ds27 (E), 5'Ovg ds27 (F) with and without 0.4 mM ATP and NaCl (0- 100 mM).
(**G**) The RNA K_D values from the fluorescence polarization titrations in panels A-D for 5'OH and 5'Ovg ds27 RNA.

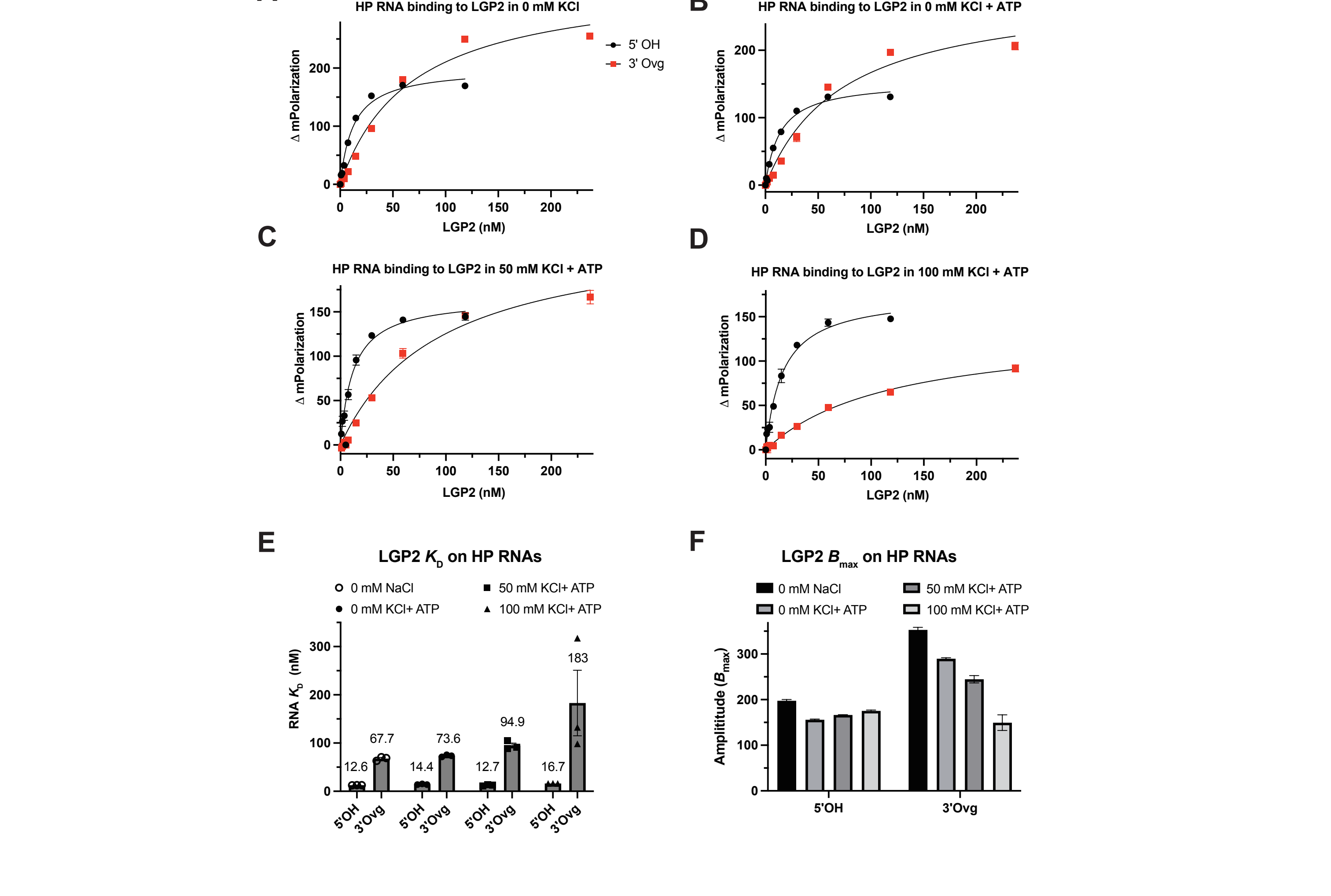

Supplemental Figure 4. LGP2 shows a preference for binding to blunt-ended dsRNA (KCl). (related to Figure 4).
(**A** - **D**) Fluorescence polarization titrations of LGP2 binding to HP RNA with blunt or overhang end modifications. (A) no ATP, (B-D) 400 mM ATP in no KCl (B), 50 mM KCl (C), and 100 mM KCl (D) in buffer A.
(**E**) The equilibrium dissociation constant (K_D) of LGP2 for the HP RNA (A-D) was assessed by fitting the fluorescence polarization titration curves and presented in bar chart form. The mean values and error bars of three replicates are shown.
(**F**) Fluorescence polarization titration amplitudes (B_{max}) of LGP2 binding to 5'OH and 3'Ovg HP RNAs with and without 400 mM ATP and KCl (0- 100 mM).

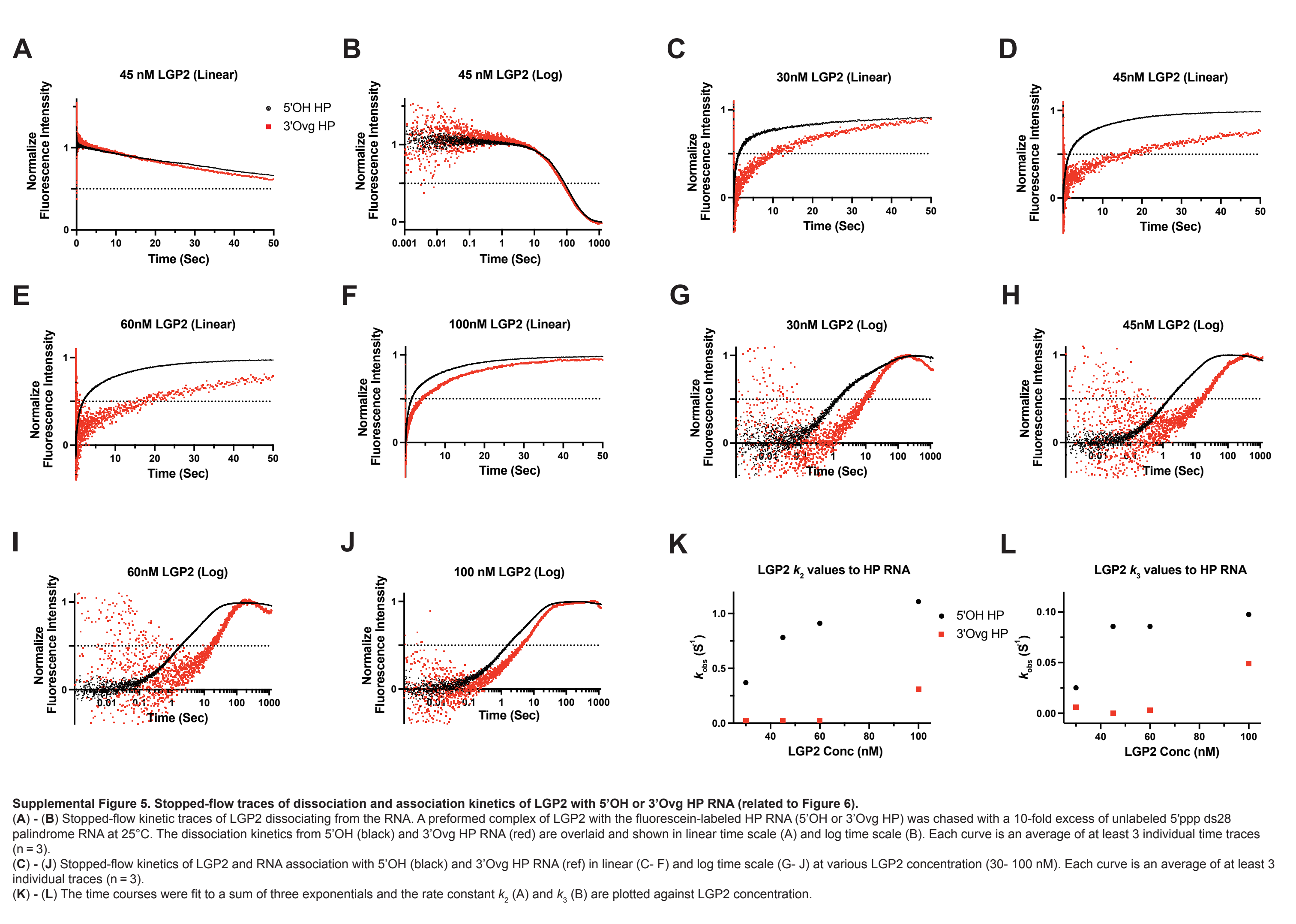

Supplemental Figure 5. Stopped-flow traces of dissociation and association kinetics of LGP2 with 5'OH or 3'Ovg HP RNA (related to Figure 6).
(**A**) - (**B**) Stopped-flow kinetic traces of LGP2 dissociating from the RNA. A preformed complex of LGP2 with the fluorescein-labeled HP RNA (5'OH or 3'Ovg HP) was chased with a 10-fold excess of unlabeled 5'ppp ds28 palindrome RNA at 25°C. The dissociation kinetics from 5'OH (black) and 3'Ovg HP RNA (red) are overlaid and shown in linear time scale (A) and log time scale (B). Each curve is an average of at least 3 individual time traces (n = 3).
(**C**) - (**J**) Stopped-flow kinetics of LGP2 and RNA association with 5'OH (black) and 3'Ovg HP RNA (ref) in linear (C- F) and log time scale (G- J) at various LGP2 concentration (30- 100 nM). Each curve is an average of at least 3 individual traces (n = 3).
(**K**) - (**L**) The time courses were fit to a sum of three exponentials and the rate constant k_2 (A) and k_3 (B) are plotted against LGP2 concentration.

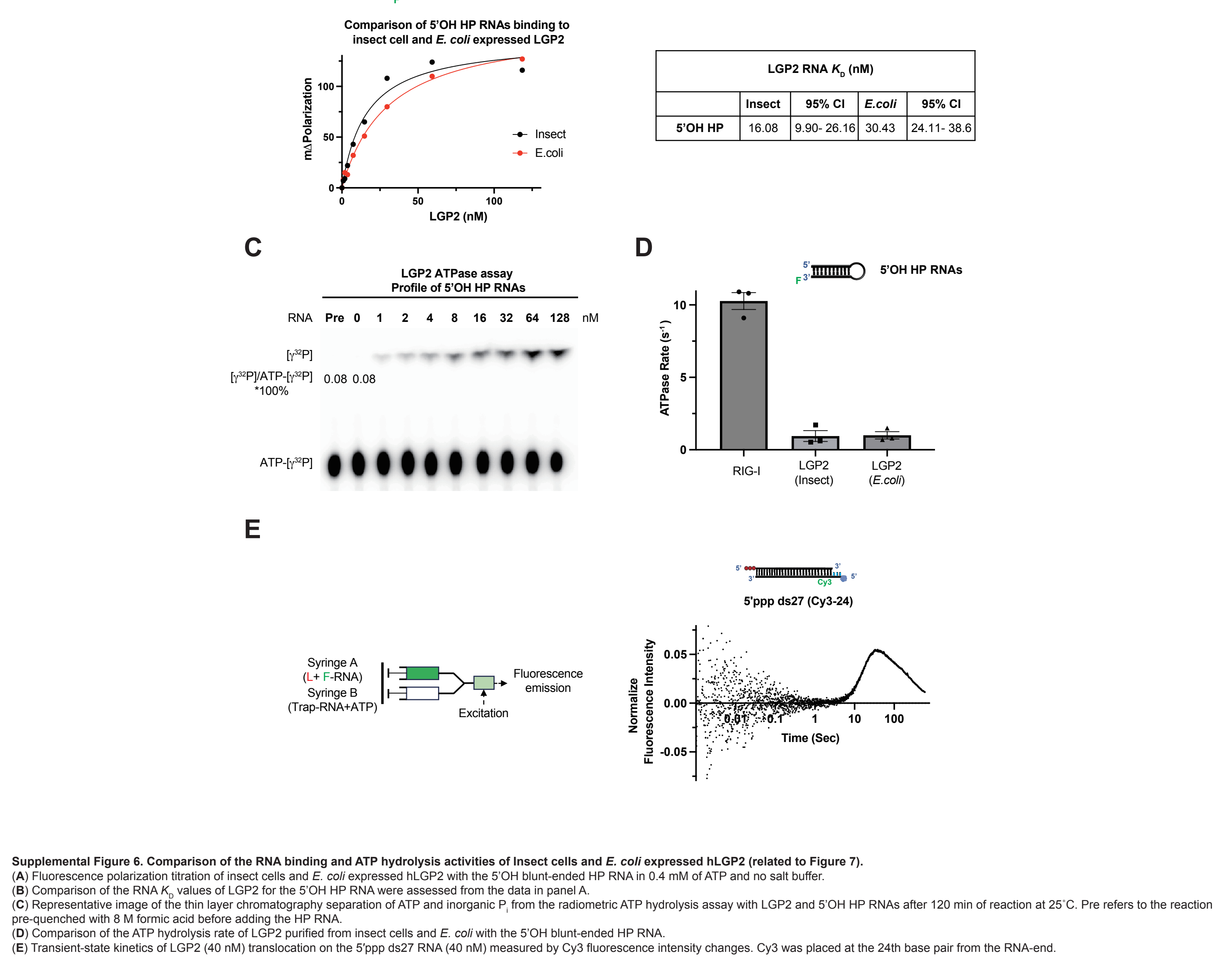

Supplemental Figure 6. Comparison of the RNA binding and ATP hydrolysis activities of Insect cells and *E. coli* expressed hLGP2 (related to Figure 7).
(**A**) Fluorescence polarization titration of insect cells and *E. coli* expressed hLGP2 with the 5'OH blunt-ended HP RNA in 0.4 mM of ATP and no salt buffer.
(**B**) Comparison of the RNA K_D values of LGP2 for the 5'OH HP RNA were assessed from the data in panel A.
(**C**) Representative image of the thin layer chromatography separation of ATP and inorganic P_i from the radiometric ATP hydrolysis assay with LGP2 and 5'OH HP RNAs after 120 min of reaction at 25˚C. Pre refers to the reaction pre-quenched with 8 M formic acid before adding the HP RNA.
(**D**) Comparison of the ATP hydrolysis rate of LGP2 purified from insect cells and *E. coli* with the 5'OH blunt-ended HP RNA.
(**E**) Transient-state kinetics of LGP2 (40 nM) translocation on the 5'ppp ds27 RNA (40 nM) measured by Cy3 fluorescence intensity changes. Cy3 was placed at the 24th base pair from the RNA-end.

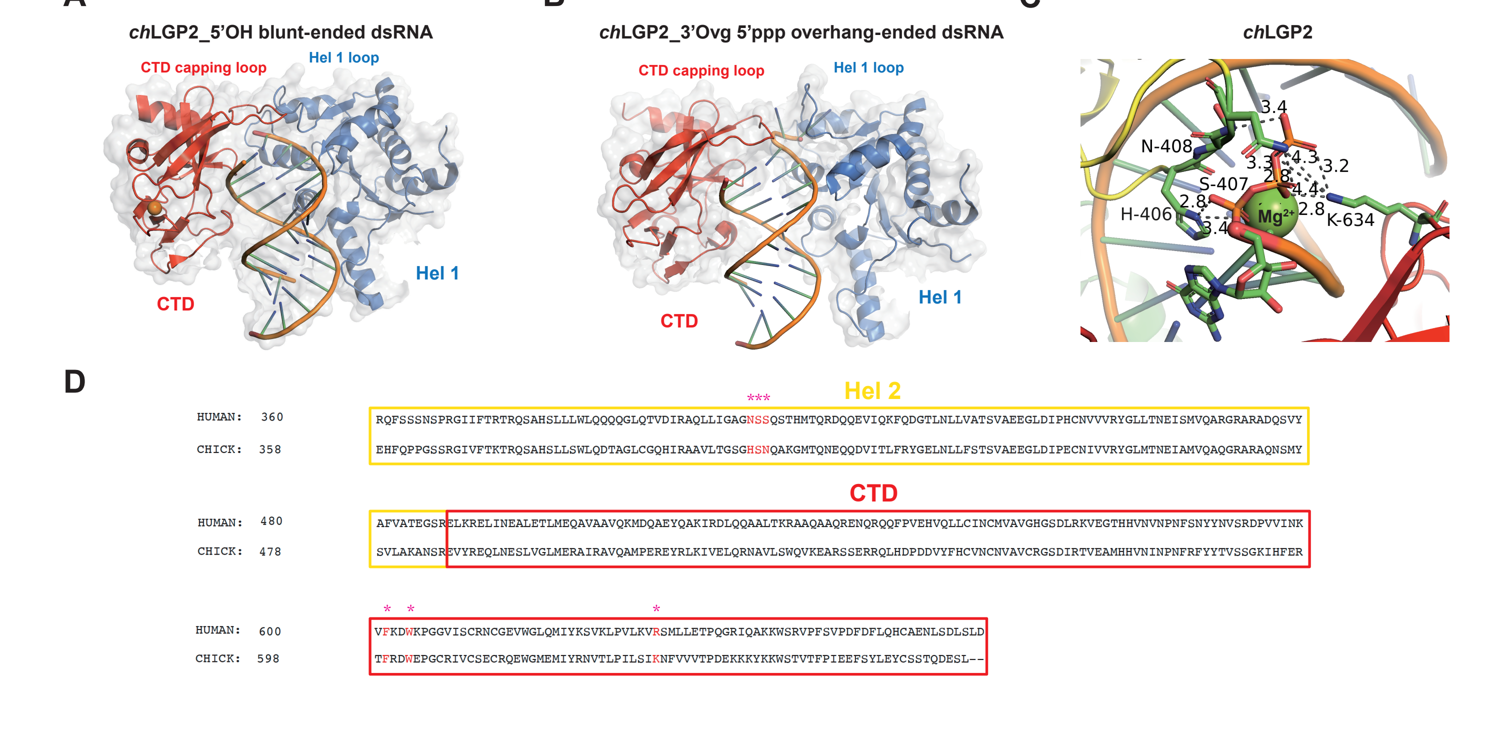

Supplemental Figure 7. Structural analysis of chLGP2 with blunt-end and overhang RNAs (related to discussion).
(**A**) - (**B**) *ch*LGP2 bound to blunt-end dsRNA (PDB: 5JBJ). The capping loop from the CTD (red) and Hel 1 loop from the Hel1 domain (blue) interact with the blunt-end bp.
(**C**) *ch*LGP2 bound to 5'ppp 3'-GG overhang dsRNA (PDB: 5JBG) shows the disengagement of the Hel 1 loop due to the 3'-GG overhang.
(**D**) Alignment of amino acid sequences of the HEL2 and CTD domains of human and chicken LGP2. Red asterisks are the amino acids mentioned in the discussion.

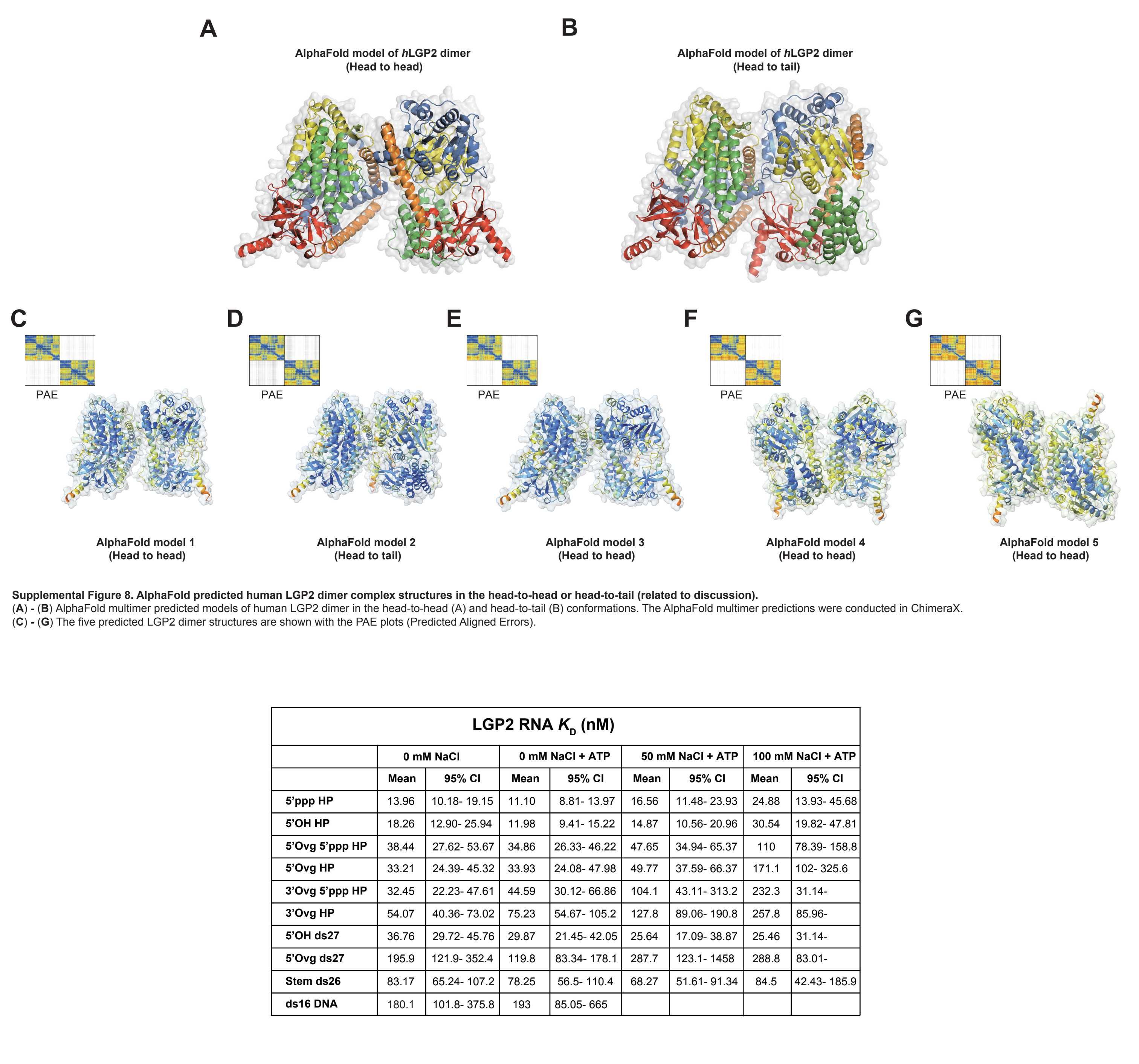

Supplemental Figure 8. AlphaFold predicted human LGP2 dimer complex structures in the head-to-head or head-to-tail (related to discussion).
(**A**) - (**B**) AlphaFold multimer predicted models of human LGP2 dimer in the head-to-head (A) and head-to-tail (B) conformations. The AlphaFold multimer predictions were conducted in ChimeraX.
(**C**) - (**G**) The five predicted LGP2 dimer structures are shown with the PAE plots (Predicted Aligned Errors).

LGP2 RNA K_D (nM)								
	0 mM NaCl		0 mM NaCl + ATP		50 mM NaCl + ATP		100 mM NaCl + ATP	
	Mean	95% CI	Mean	95% CI	Mean	95% CI	Mean	95% CI
5'ppp HP	13.96	10.18- 19.15	11.10	8.81- 13.97	16.56	11.48- 23.93	24.88	13.93- 45.68
5'OH HP	18.26	12.90- 25.94	11.98	9.41- 15.22	14.87	10.56- 20.96	30.54	19.82- 47.81
5'Ovg 5'ppp HP	38.44	27.62- 53.67	34.86	26.33- 46.22	47.65	34.94- 65.37	110	78.39- 158.8
5'Ovg HP	33.21	24.39- 45.32	33.93	24.08- 47.98	49.77	37.59- 66.37	171.1	102- 325.6
3'Ovg 5'ppp HP	32.45	22.23- 47.61	44.59	30.12- 66.86	104.1	43.11- 313.2	232.3	31.14-
3'Ovg HP	54.07	40.36- 73.02	75.23	54.67- 105.2	127.8	89.06- 190.8	257.8	85.96-
5'OH ds27	36.76	29.72- 45.76	29.87	21.45- 42.05	25.64	17.09- 38.87	25.46	31.14-
5'Ovg ds27	195.9	121.9- 352.4	119.8	83.34- 178.1	287.7	123.1- 1458	288.8	83.01-
Stem ds26	83.17	65.24- 107.2	78.25	56.5- 110.4	68.27	51.61- 91.34	84.5	42.43- 185.9
ds16 DNA	180.1	101.8- 375.8	193	85.05- 665				

Supplemental Table 1. LGP2 K_D values with different dsRNAs and DNA.
The RNA K_D values of LGP2 in different NaCl conditions were measured using fluorescence polarization titration assay (Figures 4 and 5). The mean K_D values and the 95% confidence intervals (CI) of three replicates.

LGP2-RNA dissociation kinetics				
	A1 (fluorescence intensity)	k_1 (s^{-1})	A2 (fluorescence intensity)	k_2 (s^{-1})
5'OH HP	0.27 ± 0.03 (70.5%)	0.05 ± 0.03	0.11 ± 0.03 (29.5%)	0.02 ± 0.004
3'Ovg HP	0.02 ± 0.01 (19%)	0.53 ± 0.0006	0.07 ± 0.008 (81%)	0.008 ± 0.001

Supplemental Table 2. Dissociation kinetics of 5'OH and 3'Ovg HP RNAs from LGP2 in 100 mM NaCl without ATP.
The dissociation kinetics of LGP2 (Figure 6) from the specified RNA were fit to a sum of two exponentials ($A_1e^{-k1t}+ A_2e^{-k2t}+ c$). The observed amplitudes (A) and their respective dissociation rates (k) are presented.

LGP2-RNA association kinetics						
[LGP2]	A1 (fluorescence intensity)	k_1 (s^{-1})	A2 (fluorescence intensity)	k_2 (s^{-1})	A3 (fluorescence intensity)	k_3 (s^{-1})
5'OH HP						
30 nM	-0.11 ± 0.02 (39%)	2.22 ± 0.43	-0.10 ± 0.02 (34.8%)	0.37 ± 0.10	-0.07± 0.01 (26.2%)	0.03 ± 0.01
45 nM	-0.05 ± 0.02 (15.2%)	4.26 ± 1.23	-0.15 ± 0.01 (41.5%)	0.78 ± 0.13	-0.15 ± 0.01 (43.3%)	0.09 ± 0.01
60 nM	-0.04 ± 0.01 (10.7%)	6.27 ± 1.95	-0.13 ± 0.01 (40.4%)	0.91 ± 0.12	-0.16 ± 0.01 (48.9%)	0.09 ± 0.004
100 nM	-0.03 ± 0.004 (9.8%)	11.82 ± 2.90	-0.14 ± 0.004 (42.2%)	1.11 ± 0.09	-0.16 ± 0.004 (48%)	0.10 ± 0.004
3'Ovg HP						
30 nM	-0.02 ± 0.01 (29.3%)	0.42 ± 0.20	-0.04 ± 0.04 (52.5%)	0.03 ± 0.02	0.01 ± 0.04 (18.2%)	0.01 ± 0.02
45 nM	-0.01 ± 0.003 (18.6%)	0.76 ± 0.82	-0.02 ± 0.01 (65%)	0.03 ± 0.01	0.01 ± 7.33 (16.3%)	-0.0001 ± 0.13
60 nM	-0.003 ± 0.003 (1.8%)	0.97 ± 1.85	-0.20 ± 0.02 (96.6%)	0.02 ± 0.02	0.003 ± 0.01 (1.6%)	0.003 ± 0.04
100 nM	-0.02 ± 0.03 (14.7%)	1.46 ± 1.56	-0.03 ± 0.02 (24.2%)	0.31 ± 0.38	-0.08 ± 0.02 (61.1%)	0.05 ± 0.01

Supplemental Table 3. Association kinetics of 5'OH and 3'Ovg HP RNAs with LGP2 in 100 mM NaCl without ATP.
The association kinetics of LGP2 (30-100 nM) with the specified RNA in 100 mM NaCl were fit to a sum of three exponentials ($A_1e^{-k1t}+ A_2e^{-k2t}+ A_3e^{-k3t}+ c$). The observed amplitude (A) and their respective rate constants (k) are presented.

ATPase rate constants (s^{-1})		
	LGP2	RIG-I
5'ppp HP	1.3 ± 0.2	12.2 ± 1.2
5'OH HP	1.0 ± 0.3	10.3 ± 0.6
5'Ovg 5'ppp HP	1.6 ± 0.5	5.1 ± 0.1
5'Ovg HP	1.4 ± 0.2	3.8 ± 1.0
3'Ovg 5'ppp HP	1.3 ± 0.2	6.5 ± 0.5
3'Ovg HP	1.3 ± 0.2	6.3 ± 0.2
Stem ds26	0.7 ± 0.1	N.D.

Supplemental Table 4. ATPase turnover rate of RIG-I and *E. coli* expressed LGP2 with different RNAs.
The ATPase turnover rate of RIG-I and *E. coli* expressed LGP2 was determined at increasing of RNA concentration. The maximal ATPase rate (moles of ATP hydrolyzed per mole of protein per second) at saturating RNA concentration is shown (Figure 7A and B). N.D. is non-detectable.

Oligo, Reagent or Resource			
**Oligonucleotides**			
	Sequence	Source	Identifier
5'ppp HP RNA	5'-pppGAAUAUAAUAGUGAUAUUAUAUUC(FAM)-3'	TriLink	Devarkar *et al.*, (2016)
5'OH HP RNA	5'-GAAUAUAAUAGUGAUAUUAUAUUC(FAM)-3'	TriLink	Devarkar *et al.*, (2016)
5'Ovg 5'ppp HP RNA	5'-pppAUGAAUAUAAUAGUGAUAUUAUAUUC(FAM)-3'	Biosynthesis	This paper
5'Ovg HP RNA	5'-AUGAAUAUAAUAGUGAUAUUAUAUUC(FAM)-3'	Biosynthesis	This paper
3'Ovg 5'ppp HP RNA	5'-pppGAAUAUAAUAGUGAUAUUAUAUUCGG(FAM)-3'	Biosynthesis	This paper
3'Ovg HP RNA	5'-GAAUAUAAUAGUGAUAUUAUAUUCGG(FAM)-3'	Biosynthesis	This paper
Complementary ss26 RNA chi	5'-dCdGdAdTdGGAUACUAACUAUCAGGAdCdG(U-FAM)dA-3'	Horizon	This paper
ss26 RNA chi	5'-dAdTdAdCdGUCCUGAUAGUUAGUAUCdCdAdTdC-3'	Dharmacon	Devarkar *et al.*, (2018)
Complementary ss27 RNA	5'-Bi-CGAUGGAUACUAACUAUCAGGACGUA(U-FAM)-3'	Dharmacon	Devarkar *et al.*, (2018)
5'ppp ss27 RNA	5'-pppAUACGUCCUGAUAGUUAGUAUCCAUCG-3'	Dharmacon	Devarkar *et al.*, (2018)
5'OH ss27 RNA	5'-AUACGUCCUGAUAGUUAGUAUCCAUCG-3'	Dharmacon	Devarkar *et al.*, (2016)
Cap-0 ss27 RNA	5'-(m7G)-pppAUACGUCCUGAUAGUUAGUAUCCAUCG-3'	Biosynthesis	Devarkar *et al.*, (2016)
Cap-1 ss27 RNA	5'-(m7G)-pppmAUACGUCCUGAUAGUUAGUAUCCAUCG-3'	Biosynthesis	Devarkar *et al.*, (2016)
5'OH ss29 (For 5'Ovg) RNA	5'-CUAUACGUCCUGAUAGUUAGUAUCCAUCG- 3'	Biosynthesis	Schweibenz *et al.*, (2022)
Complementary ss27 RNA	5'-Bi-CGAUGGAUACUAACUAUCAGGACGUAU-3' DY547	Dharmacon	Devarkar *et al.*, (2018)
Complementary ss27 methylated RNA	5'-Bi-CGAUGGmAmUmAmCmUAACUAUCAGGACGUAU-3' DY547	Dharmacon	Devarkar *et al.*, (2018)
5'ppp ss27 methylated RNA	5'-pppAUACGUCCUGAUAGmUmUmAmGmUAUCCAUCG-3'	Biosynthesis	Devarkar *et al.*, (2018)
complementary ss30 RNA chi (intCy3 at 24 nt from 3' end)	5'-Bi-dGdCdTCGA(U-Cy3)GGAUACUAACUAUCAGGACGUAU-3'	TriLink	Devarkar *et al.*, (2018)
Complementary ss39 RNA	5'-Desthiobiotin dT(dT-FAM)dTCCAAACAAACCAACCACAAACACACAACAACCACCAACU-3'	TriLink	Schweibenz *et al.*, (2022)
5'ppp ss39 RNA(for 5'ppp ds39)	5'-pppAGUUGGUGGUUGUUGUGUGUUUGUGGUUGGUUUGUUUGG-3'	*In vitro* transcription	Schweibenz *et al.*, (2022)
5'OH ss41 RNA (For 5'OH 5'Ovg)	5'-AGAGUUGGUGGUUGUUGUGUGUUUGUGGUUGGUUUGUUUGG-3'	Dharmacon	Schweibenz *et al.*, (2023)
5' ppp ss39 DNA transcription template strand	5'-CCAAACAAACCAACCACAAACACACAACAACCACCAACTAATAGTGAGTCGTATTACTG-3'	IDT	Schweibenz *et al.*, (2022)
5' ppp ss39 DNA transcription template strand	5'-CAGTAATACGACTCACTATTAGTTGGTGGTTGTTGTGTGTTTGTGGTTGGTTTGTTTGG-3'	IDT	Schweibenz *et al.*, (2022)
Complementary ss28 RNA	5'-GGAACUUAUCUAGUACUAGAUAAGUUCC-3'	*In vitro* transcription	This paper
5'ppp ss28 RNA (For 5'ppp ds28 palindrome RNA)	5'-pppGGAACUUAUCUAGUACUAGAUAAGUUCC-3'	*In vitro* transcription	This paper
5' ppp ss28 DNA transcription template strand	5'-GGAACTTATCTAGTACTAGATAAGTTCC-3'	IDT	This paper
5' ppp ss28 DNA transcription template strand	5'-GGAACTTATCTAGTACTAGATAAGTTCC-3'	IDT	This paper
Complementary ss16 DNA	5'-ATAACTCTTGAACAC(T-FAM)-3'	IDT	Johnson *et al.*, (2023)
ss16 DNA (For ds16 DNA)	5'-AGTGTTCAAGAGTTAT-3'	IDT	Johnson *et al.*, (2023)
**Recombinant DNA**			
pET28b 6xHis-SUMO-LGP2		This paper	This paper
pBacPAK9 6xHis-LGP2		This paper	This paper
pBacPAK6 (Bsu36 I digest)		Takara	Cat. # 631401
**Chemicals, Kit, and others**			
ATP, [γ-32P]		Perkin Elmer	Cat. # NEG002A250U
In-Fusion HD Cloning Kit		Takara	Cat. # 639648
TLC PEI Cellulose F		Millipore	Cat.# 105579
Monovalent streptavidin		Dr. Mark Howarth	University of Oxford
**Experimental models: Cell lines**			
SF9 Cell Line		Dr. Y. Whitney Yin	The University of Texas Medical Branch
**Software**			
ImageQuant TL 8.2		GE Healthcare Bio-Scienses AB	https://imagequanttl.software.informer.com/
Kintek Explorer Enzyme Kinetics Software		Kintek	https://www.kintekexplorer.com
GraphPad Prism 9.0		GraphPad	https://www.graphpad.com/
UCSF ChimeraX		Pettersen *et al.*, (2004)	https://www.cgl.ucsf.edu/chimerax/

Supplemental Table 5. Oligonucleotide sequences, recombinant DNA, reagents, cell lines, and software used in this study.
DNA nucleotides are indicated with a preceding "d" in RNA oligonucleotides. "T-FAM" indicates a T labeled with fluorescein. "FAM" indicates fluorescein. "U-Cy3" indicates a U labeled with Cy3. "Cy3" indicates Cyanine dye 3.
